# A Sesquiterpenoid from *Schizophyllum commune* Protects C17.2 Neural Stem Cells Against Oxidative Stress Through Modulation of GPCR-Associated Signaling Pathways

**DOI:** 10.3390/ijms27177974

**Published:** 2026-09-07

**Authors:** Lu Li, Guowei Zou, Qiaona Wang, Haitao Jiang, Xianghua Wu, Junli Zhao, Honglin Zhang, Xiaoping Wang, Shengjie Li

**Affiliations:** School of Food Science, Nanjing Xiaozhuang University, Nanjing 211171, China; 232702032@njnu.edu.cn (L.L.); 255211003@njxzc.edu.cn (G.Z.); 202311530005@nuist.edu.cn (Q.W.); 2005026@njxzc.edu.cn (H.J.); xhw610@163.com (X.W.); zhaojl520@njxzc.edu.cn (J.Z.); 2004030@njxzc.edu.cn (H.Z.); wangxpb@126.com (X.W.)

**Keywords:** *Schizophyllum commune*, oxidative stress, neuroprotection, C17.2 cells, GPCR, PKA signaling

## Abstract

The chemical constituents and neuroprotective potential of *Schizophyllum commune* remain insufficiently characterized. Therefore, we isolated and identified five secondary metabolites (**A**–**E**) from *S. commune*, and investigated their neuroprotective activities and underlying mechanisms. Compound **C** showed the most significant protective effect against H_2_O_2_-induced cytotoxicity in C17.2 neural stem cells. Spectroscopy was used to structurally characterize the isolated compounds. Using RNA sequencing (RNA-seq), compound **C** was found to significantly modulate genes associated with G protein-coupled receptor (GPCR)-related signaling pathways and neuroactive ligand–receptor interactions. Differentially expressed genes, including *Adora2a*, *S1pr1*, *Adm*, *Tbxa2r*, and *Grin3b*, were validated using quantitative real-time PCR. They are associated with GPCR-related signaling and neurotransmission pathways, consistent with the RNA-seq data. As indicated by the functional enrichment analysis, compound **C** may regulate neuronal stress responses through GPCR-associated signaling networks. In the Western blot, compound **C** markedly attenuated H_2_O_2_-induced protein kinase A (PKA) C phosphorylation without altering total PKA C expression, suggesting that modulation of the cAMP/PKA signaling pathway may contribute to the neuroprotective effects of compound **C**. Collectively, compound **C** may exert neuroprotective effects against oxidative stress-induced neuronal injury, potentially through the modulation of GPCR-mediated PKA signaling. *S. commune* is a promising natural bioactive compound source for further development in neuroprotective research.

## 1. Introduction

Neurodegenerative disorders are characterized by the progressive loss of neuronal structure and function, and oxidative stress is a central pathological mechanism contributing to neuronal injury. Excessive production of reactive oxygen species (ROS) leads to lipid peroxidation, mitochondrial dysfunction, and activation of apoptotic signaling pathways, ultimately resulting in neuronal cell death [1]. Among various experimental models, H_2_O_2_-induced oxidative stress in neuronal cells is widely used to simulate pathological conditions associated with neurodegeneration. Large-scale transcriptional alterations, including upregulation of pro-oxidant genes and inflammatory mediators, are induced in this model, thereby providing a robust platform for investigating neuroprotective mechanisms [1,2].

Oxidative stress is closely linked to inflammatory responses in neuronal systems [3]. H_2_O_2_ exposure activates multiple signaling pathways, including TNF, IL-17, and Toll-like receptor (TLR) signaling pathways, which collectively contribute to neuroinflammation and cellular damage [4,5].

G protein-coupled receptors (GPCRs) are the largest family of membrane receptors in the nervous system that play essential roles in regulating neuronal excitability, synaptic transmission, and survival [6]. Dysregulation of GPCR-mediated signaling is implicated in oxidative stress-induced neuronal injury and neurodegenerative diseases [7]. Among downstream effectors of GPCR signaling, the cyclic adenosine monophosphate (cAMP)/protein kinase A (PKA) pathway is a key regulator of intracellular signal transduction. PKA activation modulates neuronal survival, gene expression, and synaptic plasticity under stress conditions [8]. However, excessive or dysregulated activation of the cAMP/PKA signaling cascade may also contribute to cellular dysfunction under oxidative stress conditions, indicating a context-dependent role in neuronal fate determination [9].

Natural products derived from fungi and medicinal plants are important sources of neuroprotective agents due to their multi-target regulatory properties [10,11]. These compounds often exert antioxidant, anti-inflammatory, and signaling-modulatory effects in neuronal systems [12,13]. *Schizophyllum commune*, a widely distributed edible and medicinal fungus, contains diverse bioactive metabolites [14,15]. However, its chemical constituents and neuroprotective potential remain insufficiently characterized, particularly in oxidative stress-induced neuronal injury.

In the present study, five compounds were isolated from *S. commune*, and their neuroprotective effects were evaluated using an H_2_O_2_-induced oxidative stress model in C17.2 neural stem cells. Among them, compound **C** exhibited the most potent neuroprotective activity and was therefore selected for further investigation to elucidate its underlying molecular mechanisms, with particular emphasis on GPCR-associated signaling and the PKA pathway.

## 2. Results

### 2.1. Isolation and Structural Elucidation of Compounds ***A**–**E***

**Figure 1:**
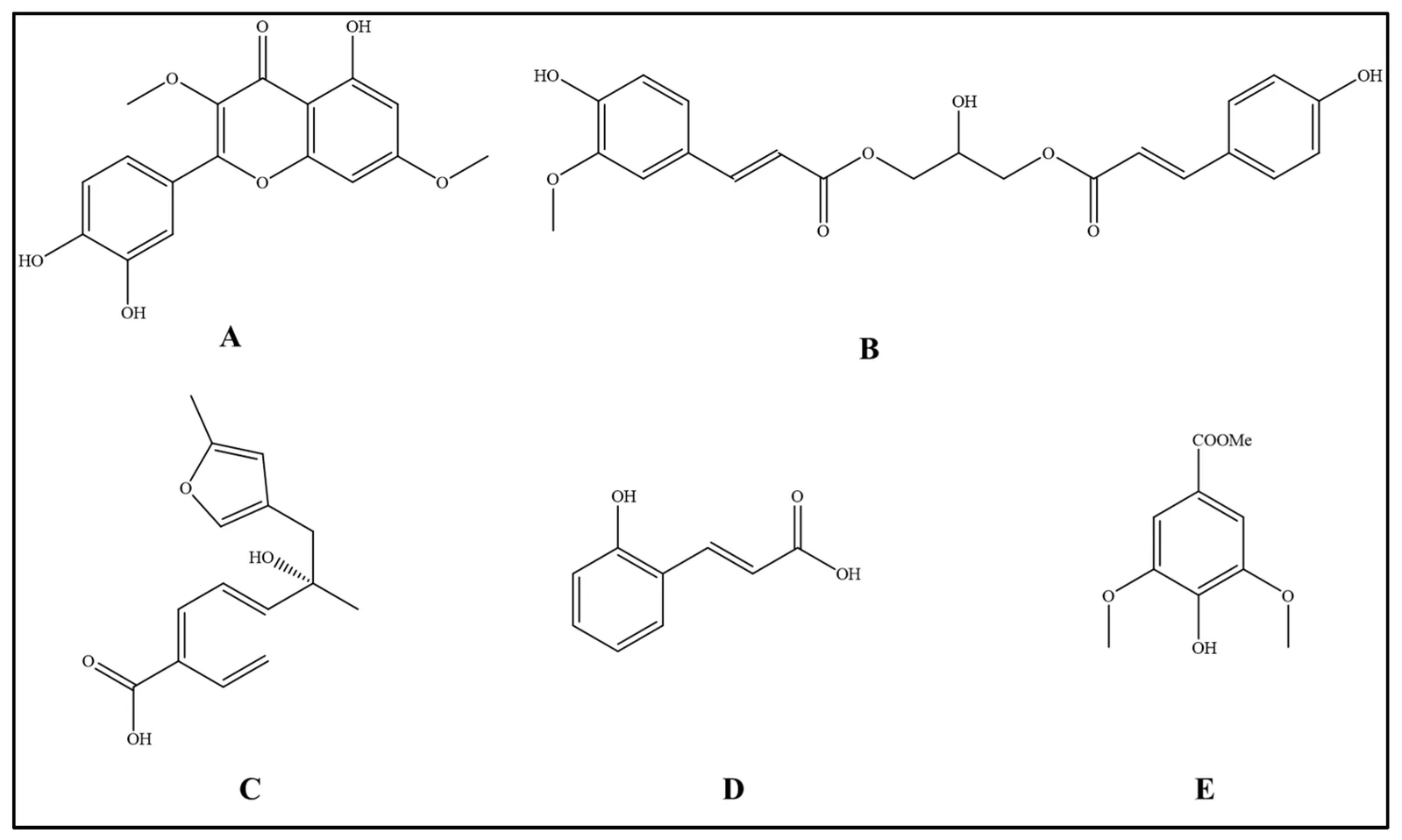
Structures of compounds **A**–**E** derived from *S. commune*. (**A**) Compound **A** (C_17_H_14_O_7_, 15.6 mg, 0.156% of the methanol extract), identified as a flavonoid derivative bearing two methoxy substituents. (**B**) Compound **B** (C_22_H_22_O_8_, 6.8 mg, 0.068% of the methanol extract), identified as a phenylpropanoid glyceride containing feruloyl and *p*-coumaroyl moieties. (**C**) Compound **C** (C_15_H_16_O_4_, 13.6 mg, 0.136% of the methanol extract), identified as a sesquiterpenoid featuring an epoxide bridge and a carboxyl group. (**D**) Compound **D** (C_9_H_8_O_3_, 3.0 mg, 0.030% of the methanol extract), identified as a phenolic acid derivative. (**E**) Compound **E** (C_10_H_12_O_5_, 20.9 mg, 0.209% of the methanol extract), identified as a phenolic acid derivative. **A**–**E** represent individual subfigures corresponding to compounds **A**–**E**, respectively.

Five compounds were isolated from a methanol extract of *S. commune* fruiting bodies through activity-guided fractionation, repeated column chromatography, and semi-preparative HPLC purification. Their chemical structures were elucidated using comprehensive spectroscopic analyses, including high-resolution electrospray ionization mass spectrometry (HR-ESI-MS) and one-dimensional (1D) and two-dimensional (2D) nuclear magnetic resonance (NMR) spectroscopy, including ^1^H NMR, ^13^C NMR, distortionless enhancement by polarization transfer (DEPT), correlation spectroscopy (COSY), heteronuclear single quantum coherence (HSQC), and heteronuclear multiple bond correlation (HMBC). The obtained spectroscopic data were further compared with previously reported literature data for structural identification. The isolated compounds were identified as 3,7-dimethylquercetin (Compound **A**), 1-O-feruloyl-3-O-p-coumaroylglycerol (Compound **B**), (+)-(7S)-15-carboxy-9,13-epoxy-7-hydroxy-9,13-dehydro-ar-curcumene (Compound **C**), o-coumaric acid (Compound **D**), and methyl syringate (Compound **E**) (Figure 1).

Compound **A** (C_17_H_14_O_7_, 15.6 mg, corresponding to 0.156% of the methanol extract) was characterized as a flavonoid derivative with two methoxy substituents. Compound **B** (C_22_H_22_O_8_, 6.8 mg, corresponding to 0.068% of the methanol extract) was a phenylpropanoid glyceride containing both feruloyl and p-coumaroyl moieties. Compound **C** (C_15_H_16_O_4_, 13.6 mg, corresponding to 0.136% of the methanol extract) was a sesquiterpenoid featuring an epoxide bridge and carboxyl group, whereas compounds **D** (C_9_H_8_O_3_, 3.0 mg, corresponding to 0.030% of the methanol extract) and **E** (C_10_H_12_O_5_, 20.9 mg, corresponding to 0.209% of the methanol extract) were determined to be phenolic acid derivatives. The structures of compounds **A**–**E** were unambiguously established based on their characteristic NMR correlations and HR-MS data. Detailed spectroscopic data are provided in the Supplementary Materials (Figures S1–S14 and S19).

### 2.2. Effects of Compounds ***A**–**E*** on C17.2 Cell Viability

The cytotoxicity of compounds **A**–**E** toward C17.2 cells was initially evaluated using a CCK-8 assay. C17.2 cells were exposed to different concentrations (0.1–100 μg/mL) of each compound for 24 h.

**Figure 2:**
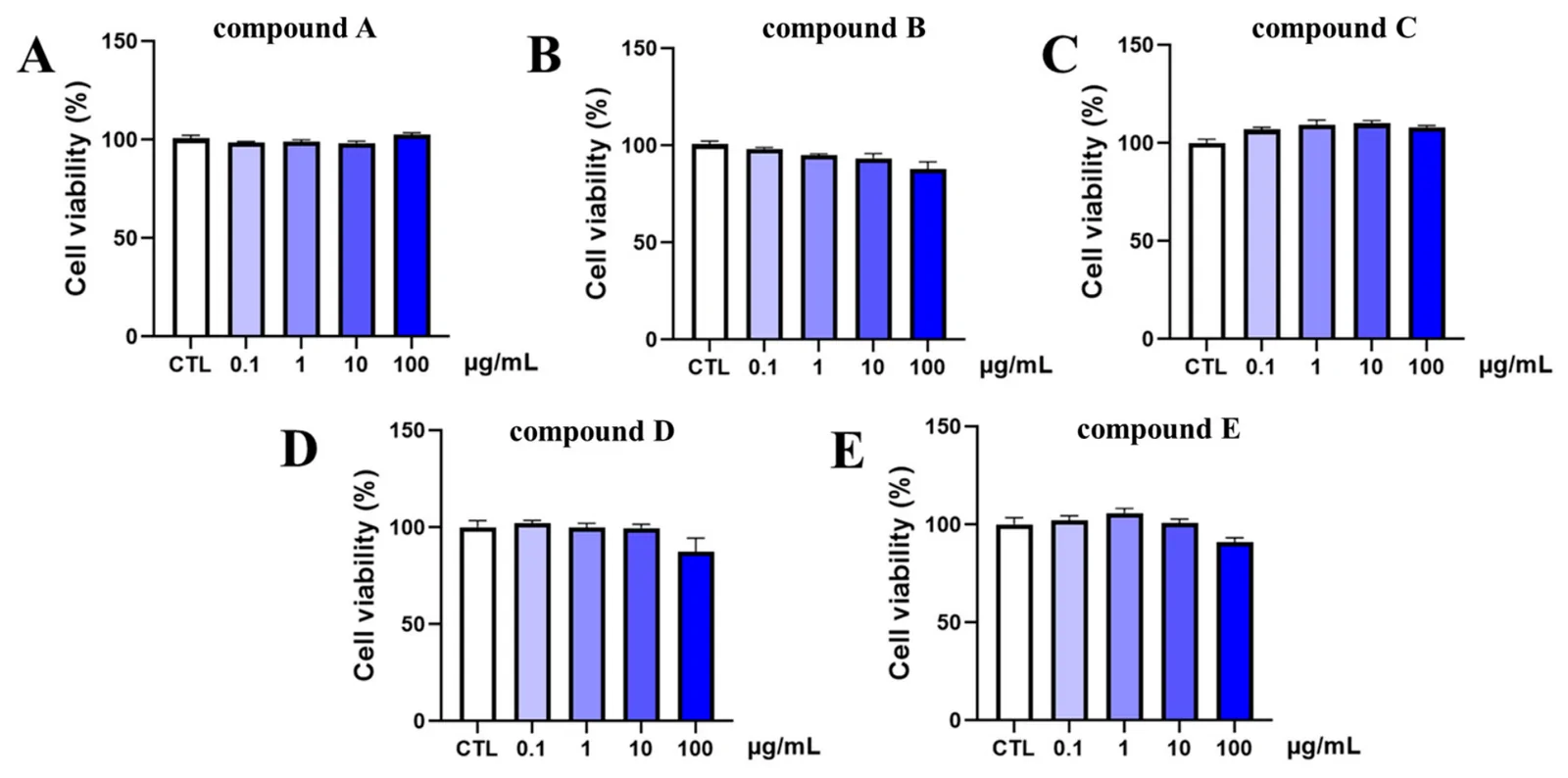
Effects of compounds **A**–**E** on C17.2 cell viability. (**A**) Effect of compound **A** on C17.2 cell viability. (**B**) Effect of compound **B** on C17.2 cell viability. (**C**) Effect of compound **C** on C17.2 cell viability. (**D**) Effect of compound **D** on C17.2 cell viability. (**E**) Effect of compound **E** on C17.2 cell viability. C17.2 cells were treated with compounds **A**–**E** at concentrations of 0.1, 1, 10, and 100 μg/mL. Each group consists of three replicates. Values are represented as mean ± standard error of mean from three independent biological experiments (*n* = 3). Statistical significance was determined using one-way ANOVA followed by Tukey’s multiple comparisons test. CTL, control.

Compound **A** did not significantly affect the viability of C17.2 cells at any tested concentration, and cell viability remained above 90% compared with that of the control group (Figure 2A). Similarly, compound **B** exhibited no obvious cytotoxic effects on C17.2 cells across the tested concentration range (Figure 2B). Cell viability in all treatment groups was comparable to that of the control group. Compound **C** also showed negligible cytotoxicity, with no significant reduction in cell viability observed following treatments at 0.1 to 100 μg/mL (Figure 2C). Similarly, treatment with compound **D** did not significantly alter C17.2 cell viability at any concentration (Figure 2D). Additionally, compound **E** displayed no detectable cytotoxicity under the experimental conditions, and cell viability remained above 90% in all treatment groups (Figure 2E).

Overall, compounds **A**–**E** exhibited low cytotoxicity toward C17.2 cells within the tested concentration range, indicating favorable cellular safety profiles. Therefore, 0.1 to 100 μg/mL was selected for subsequent evaluation of their neuroprotective effects against H_2_O_2_-induced oxidative injury.

### 2.3. Neuroprotective Effects of Compounds ***A**–**E*** Against H_2_O_2_-Induced Injury

To evaluate the neuroprotective potential of the isolated compounds, C17.2 cells were pretreated with compounds **A**–**E** at different concentrations for 24 h prior to H_2_O_2_ exposure, and cell viability was subsequently determined using the CCK-8 assay.

**Figure 3:**
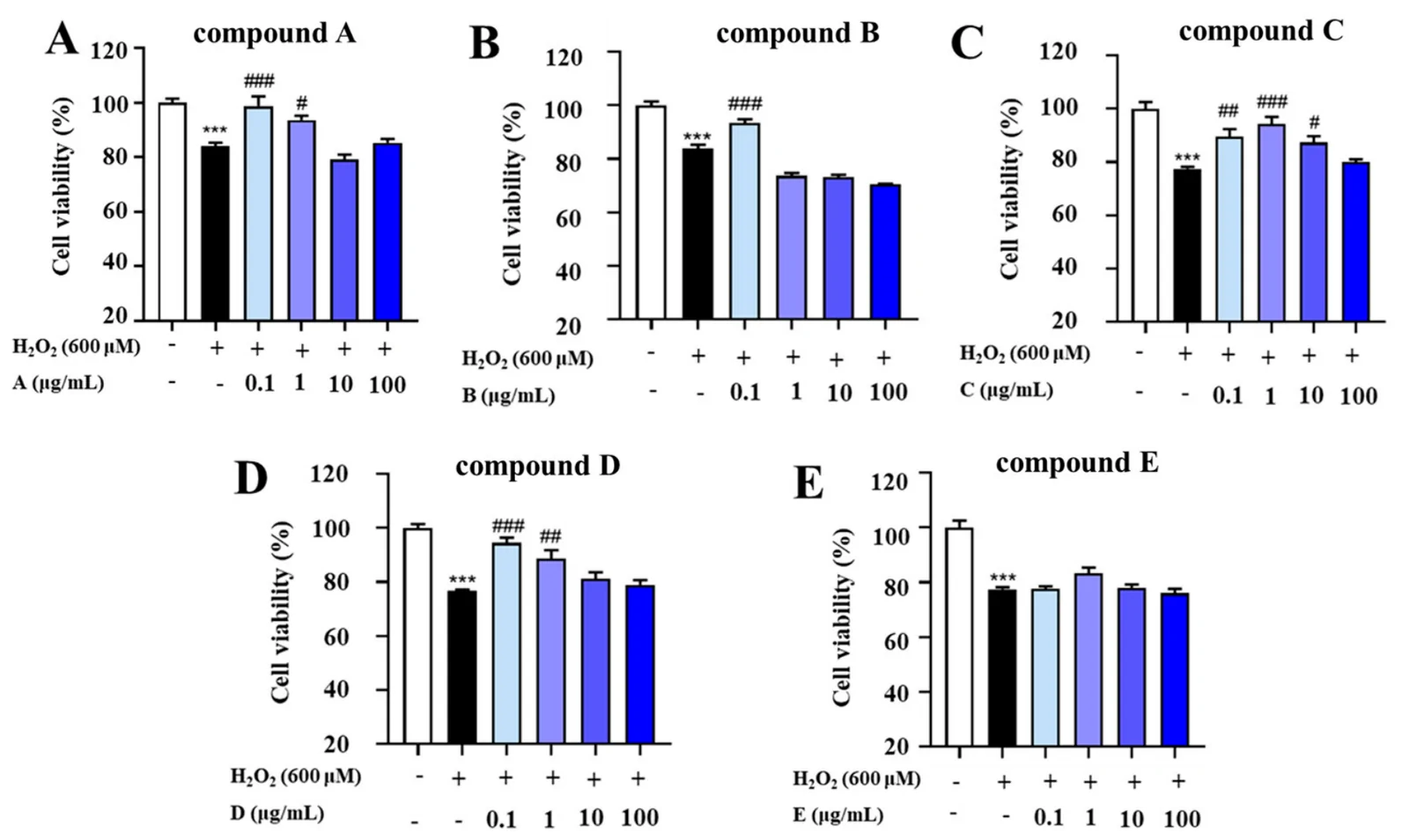
Effects of the compounds on C17.2 cell viability under H_2_O_2_−induced (600 μM) oxidative stress injury. (**A**) Effect of compound **A** on C17.2 cell viability under H_2_O_2_-induced oxidative stress injury. (**B**) Effect of compound **B** on C17.2 cell viability under H_2_O_2_-induced oxidative stress injury. (**C**) Effect of compound **C** on C17.2 cell viability under H_2_O_2_-induced oxidative stress injury. (**D**) Effect of compound **D** on C17.2 cell viability under H_2_O_2_-induced oxidative stress injury. (**E**) Effect of compound **E** on C17.2 cell viability under H_2_O_2_-induced oxidative stress injury. C17.2 cells were exposed to 600 μM H_2_O_2_ and treated with compounds **A**–**E** at concentrations of 0.1, 1, 10, and 100 μg/mL. Each group consists of three replicates. Values are represented as mean ± standard error of mean from three independent biological experiments (*n* = 3). Statistical significance was determined using one-way ANOVA followed by Tukey’s multiple comparisons test. *** (*p* < 0.001) indicates significant difference between control and H_2_O_2_. # represents a significant difference (# *p* < 0.05, ## *p* < 0.01, ### *p* < 0.001) between H_2_O_2_ and compound-H_2_O_2_ groups.

Treatment with H_2_O_2_ markedly reduced the viability of C17.2 cells compared with that of the control group (*p* < 0.001), indicating the successful establishment of the oxidative stress injury model (Figure 3A). Pretreatment with compound **A** significantly increased cell viability at 0.1 μg/mL (*p* < 0.001) and 1 μg/mL (*p* < 0.05), whereas no significant protective effect was observed at the other tested concentrations.

Compound **B** exhibited a significant neuroprotective effect at 0.1 μg/mL (*p* < 0.001). However, increasing the concentration did not further enhance its protective efficacy, and no significant differences were observed at higher concentrations (Figure 3B). Compound **C** displayed the most pronounced neuroprotective activity among all tested compounds (Figure 3C). Pretreatment with 0.1, 1, and 10 μg/mL compound **C** significantly attenuated H_2_O_2_-induced cytotoxicity (*p* < 0.05), with the maximal protective effect observed at 1 μg/mL. In contrast, treatment with 100 μg/mL compound **C** failed to improve cell viability. Compound **D** significantly restored the viability of H_2_O_2_-treated C17.2 cells at 0.1 μg/mL (*p* < 0.05) (Figure 3D). A moderate protective effect was also observed at 1 μg/mL, although this effect was weaker than that observed at the lower concentration. In contrast, compound **E** did not significantly affect cell viability at any of the tested concentrations (Figure 3E), suggesting that this compound lacked notable neuroprotective activity under the present experimental conditions.

Collectively, compounds **A**–**D** exhibited varying degrees of neuroprotective activity against H_2_O_2_-induced oxidative injury in C17.2 cells, whereas compound **E** showed no significant protective effect. Among the tested compounds, compound **C** exerted the strongest and most stable neuroprotective activity over a relatively broad concentration range. Notably, compound **C** significantly improved cell viability at concentrations of 0.1, 1, and 10 μg/mL, with the maximal protective effect observed at 1 μg/mL. Therefore, 1 μg/mL compound **C** was selected for subsequent mechanistic studies.

### 2.4. Compound ***C*** Significantly Reduced the Intracellular Oxidative Stress-Associated Fluorescent Signal Detected by DCFH-DA Staining Following H_2_O_2_ Exposure

#### 2.4.1. Compound **C** Reduces Intracellular ROS Accumulation

**Figure 4:**
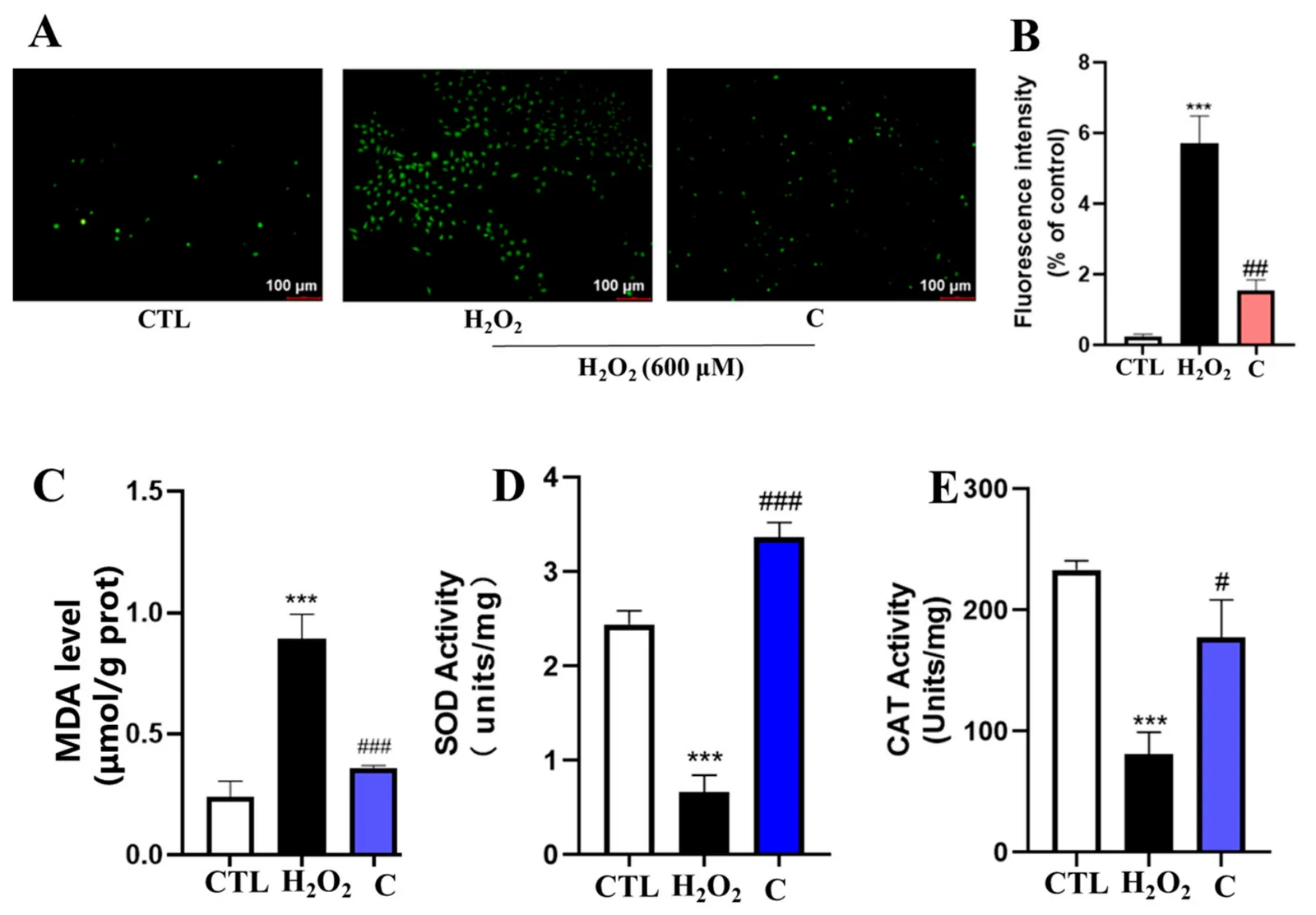
The antioxidative effect of compound **C** treatment on C17.2 cells. (**A**) Representative images of reactive oxygen species (ROS) captured (scale bar: 100 μm). (**B**) Quantitative analysis of intracellular ROS levels using Image J software. (**C**) Malondialdehyde (MDA), (**D**) superoxide dismutase (SOD), and (**E**) catalase (CAT) in treated groups. Each group consists of three replicates. Values are represented as mean ± standard error of the mean. Statistical significance was determined using one-way ANOVA followed by Tukey’s multiple comparisons test. *** (*p* < 0.001) indicates significant difference between control and H_2_O_2_. # represents a significant difference (# *p* < 0.05, ## *p* < 0.01, ### *p* < 0.001) between H_2_O_2_ and compound-H_2_O_2_ groups. CTL, control.

Intracellular ROS levels were determined using the DCFH-DA fluorescent probe. Representative fluorescence images are shown in Figure 4A. Compared with the control group, H_2_O_2_ markedly increased intracellular ROS accumulation in C17.2 cells, as evidenced by the enhanced green fluorescence intensity. In contrast, pretreatment with compound **C** significantly attenuated H_2_O_2_-induced ROS generation.

Quantitative analysis further confirmed that ROS levels in the H_2_O_2_ group were significantly higher than those in the control group (Figure 4B, *p* < 0.001). Treatment with compound **C** markedly reduced intracellular ROS levels compared with those in the H_2_O_2_-treated group (*p* < 0.001), restoring ROS production to a level close to that observed in control cells.

These results demonstrate that compound **C** effectively suppresses intracellular ROS accumulation induced by H_2_O_2_ in C17.2 cells.

#### 2.4.2. Compound **C** Improves Cellular Oxidative Stress Status

To further evaluate the antioxidant capacity of compound **C**, intracellular MDA content and activities of antioxidant enzymes, including SOD and CAT, were determined. H_2_O_2_ exposure significantly increased intracellular MDA levels compared with that in the control group (Figure 4C), indicating marked lipid peroxidation. However, compound **C** markedly reduced MDA accumulation (*p* < 0.001), and the MDA level was restored to a level comparable to that of the control group. The activity of SOD was markedly decreased in H_2_O_2_-treated cells compared with that in control cells (Figure 4D). Notably, compound **C** significantly enhanced SOD activity relative to that in the H_2_O_2_ group (*p* < 0.001), slightly exceeding that observed in the control group. Similarly, CAT activity was significantly suppressed after H_2_O_2_ stimulation (Figure 4E). Compound **C** markedly restored CAT activity compared with that in the H_2_O_2_ group (*p* < 0.001).

Collectively, these findings indicate that compound **C** effectively alleviates H_2_O_2_-induced oxidative stress by reducing lipid peroxidation and enhancing the endogenous antioxidant defense system in C17.2 cells.

### 2.5. Transcriptomic Profiling Reveals the Neuroprotective Mechanisms of Compound ***C***

#### 2.5.1. Overview of RNA Sequencing (RNA-Seq) Data and Differentially Expressed Genes (DEGs)

To investigate transcriptional alterations induced by oxidative stress and the effects of compound **C** treatment, DEGs were first identified by comparing the H_2_O_2_-treated group with the control group (H_2_O_2_ vs. Control). Subsequently, genes associated with compound **C** treatment were identified by comparing the compound **C** + H_2_O_2_-treated group with the H_2_O_2_-treated group (Compound **C** + H_2_O_2_ vs. H_2_O_2_). Genes exhibiting opposite expression patterns between these two comparisons were considered candidate genes reversed by compound **C** treatment. To elucidate the molecular mechanisms underlying the neuroprotective effects of compound **C** against H_2_O_2_-induced oxidative injury, transcriptomic sequencing was performed in control, H_2_O_2_-treated, and compound **C**-treated C17.2 cells.

**Figure 5:**
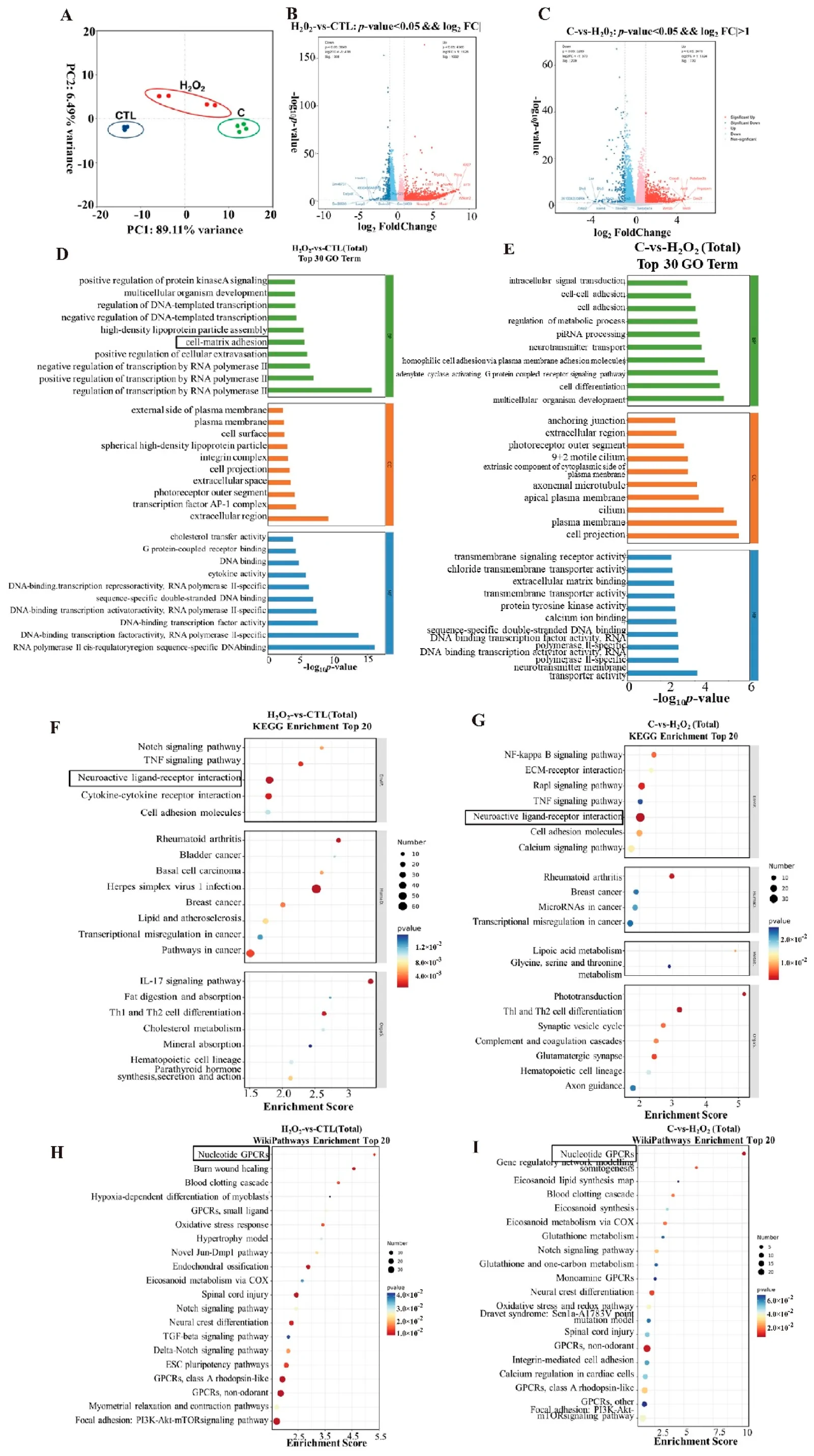
Transcriptomic analysis reveals the molecular mechanisms underlying the neuroprotective effects of compound **C** in H_2_O_2_−injured C17.2 cells. (**A**) Principal component analysis (PCA) of transcriptomic profiles from the control (CTL), H_2_O_2_, and compound **C**-treated groups. Each dot represents one biological replicate. (**B**) Volcano plot showing differentially expressed genes (DEGs) between the CTL and H_2_O_2_ groups. (**C**) Volcano plot showing DEGs between the H_2_O_2_ and compound **C**-treated groups. Red dots represent significantly upregulated genes, whereas blue dots indicate significantly downregulated genes. (**D**,**E**) Gene Ontology (GO) enrichment analyses of DEGs identified in the CTL versus H_2_O_2_ comparison (**D**) and the H_2_O_2_ versus compound **C** comparison (**E**). (**F**,**G**) Kyoto Encyclopedia of Genes and Genomes (KEGG) pathway enrichment analyses of DEGs identified in the CTL versus H_2_O_2_ comparison (**F**) and H_2_O_2_ versus compound **C** comparison (**G**). (**H**,**I**) WikiPathways enrichment analyses of DEGs identified in the CTL versus H_2_O_2_ comparison (**H**) and H_2_O_2_ versus compound **C** comparison (**I**). Data are from three independent biological experiments (*n* = 3). The size of each dot represents the number of enriched genes, and the color indicates the significance level of enrichment. CTL, control.

Principal component analysis (PCA) was conducted. There was a clear separation among the three experimental groups, while biological replicates within each group clustered closely together (Figure 5A), indicating high reproducibility and reliability of the RNA-seq data.

In the differential expression analysis, marked transcriptional alterations were observed after H_2_O_2_ exposure. Compared with the control group, a total of 8009 differentially expressed genes (DEGs) were identified in the H_2_O_2_ group, including 4360 upregulated genes and 3649 downregulated genes (Figure 5B). Furthermore, a comparison between the compound **C**-treated and H_2_O_2_ groups showed 6764 DEGs, among which 3479 genes were upregulated and 3285 genes were downregulated (Figure 5C). Collectively, these results suggest that compound **C** exerts neuroprotective effects through extensive regulation of gene expression networks.

The volcano plots further illustrated the global transcriptional alterations induced by H_2_O_2_ and extensive reversal effects exerted by compound **C** on gene expression profiles. These findings suggest that compound **C** markedly remodels the transcriptional landscape of H_2_O_2_–injured C17.2 cells and may exert neuroprotective effects through broad regulation of multiple biological processes and signaling pathways.

#### 2.5.2. Functional Enrichment Analysis of DEGs

To gain further insights into the biological significance of the identified DEGs, functional enrichment analyses were performed. Functional enrichment analyses, including Gene Ontology (GO), Kyoto Encyclopedia of Genes and Genomes (KEGG), and WikiPathways analyses, were performed to identify biological processes and signaling pathways associated with the identified DEGs.

GO enrichment analysis, which classifies genes according to their associated biological processes, cellular components, and molecular functions, was conducted. DEGs identified in the control (CTL) versus H_2_O_2_ comparison were primarily enriched in biological processes related to cell adhesion, extracellular matrix organization, and intercellular interactions (Figure 5D). In contrast, DEGs identified in the H_2_O_2_ versus compound **C** comparison were significantly enriched in processes associated with cell–cell adhesion, homophilic cell adhesion via plasma membrane adhesion molecules, and regulation of cell adhesion (Figure 5E), suggesting that compound **C** may preserve cellular integrity under oxidative stress conditions.

KEGG pathway analysis, which is widely used to identify signaling pathways and metabolic networks associated with DEGs, was conducted. H_2_O_2_-responsive genes were mainly enriched in pathways associated with neuronal signaling and cellular communication (Figure 5F). Notably, treatment with compound **C** significantly altered pathways involved in neuroactive ligand–receptor interaction and other signaling pathways associated with neuronal function (Figure 5G), indicating that modulation of neuronal signaling pathways may contribute to the neuroprotective actions of compound **C**.

WikiPathways analysis, an open and continuously curated pathway database that complements KEGG by providing additional pathway annotations, was conducted. The DEGs were significantly enriched in several signaling pathways, among which the nucleotide GPCR signaling pathway was particularly prominent in the compound **C**-treated group (Figure 5H,I). Given the well-established roles of GPCR-mediated signaling in neuronal survival and oxidative stress responses, these findings suggest that GPCR-associated pathways may participate in the neuroprotective mechanism of compound **C**.

Overall, transcriptomic analyses indicate that compound **C** exerts neuroprotective effects through coordinated regulation of cell adhesion-related processes, neuronal signaling pathways, and GPCR-mediated signaling networks.

### 2.6. Validation of DEGs by Quantitative Real-Time PCR

To validate the reliability of the RNA-seq data and further confirm the potential molecular mechanisms underlying the neuroprotective effects of compound **C**, five representative DEGs associated with GPCR signaling and neuronal function, sphingosine-1-phosphate receptor 1 (*S1pr1*), *Adora2a*, *Adm*, *Tbxa2r*, and *Grin3b*, were selected for RT-qPCR analysis.

**Figure 6:**
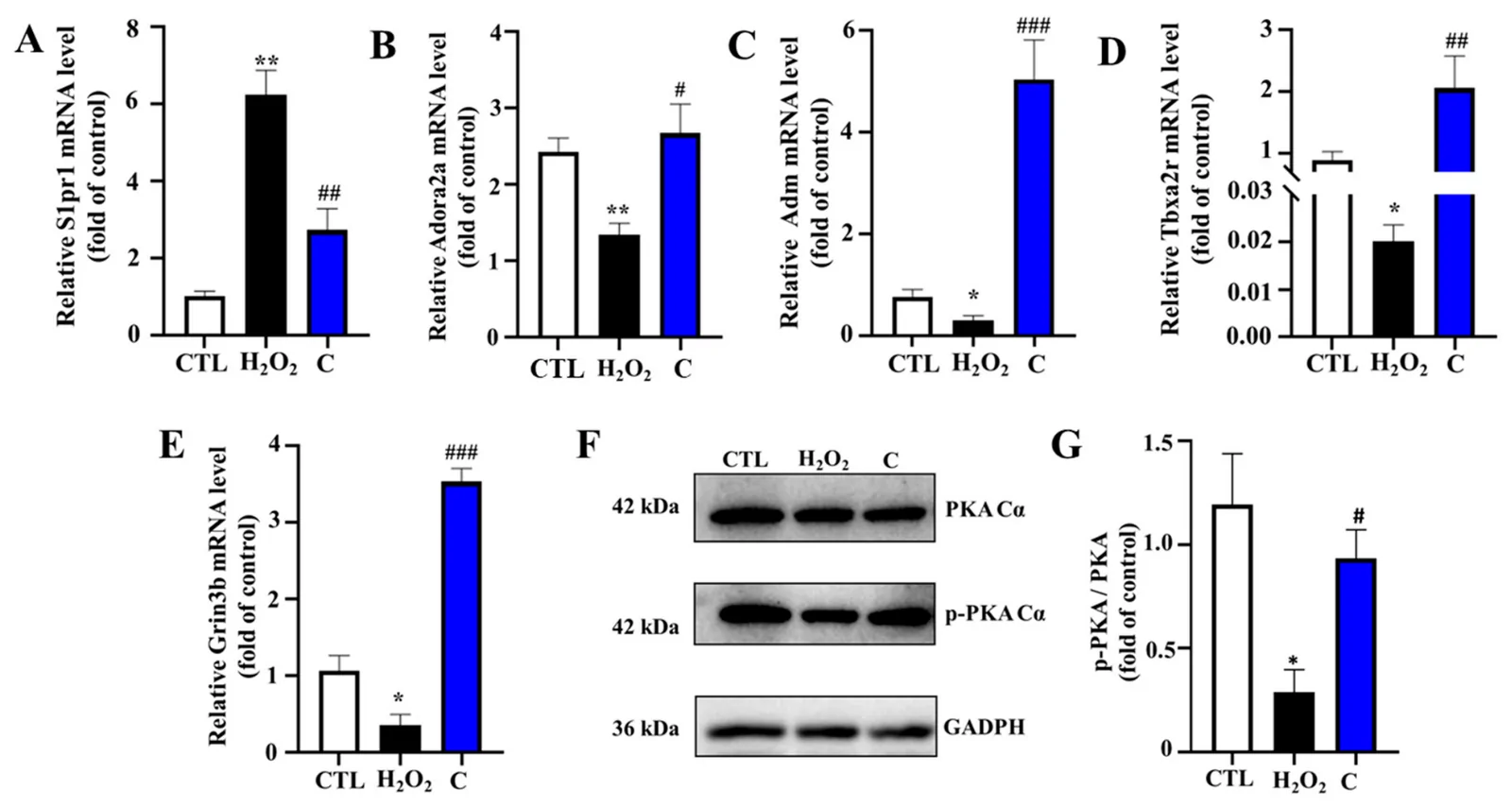
Validation of RNA-seq data using RT-qPCR. The mRNA expression levels of five representative differentially expressed genes, including (**A**) *S1pr1*, (**B**) *Adora2a*, (**C**) *Adm*, (**D**) *Tbxa2r*, and (**E**) *Grin3b*, were determined using RT-qPCR in C17.2 cells. (**F**) Representative Western blot images showing the protein expression levels of phosphorylated PKA (p-PKA Cα), total PKA C Cα and GAPDH in C17.2 cells. (**G**) Quantitative analysis of the p-PKA Cα/PKA Cα ratio. The expression trends obtained using RT-qPCR were consistent with those identified using RNA-seq analysis. Data are presented as the mean ± SEM from three independent experiments (*n* = 3). Statistical significance was determined using one-way ANOVA followed by Tukey’s multiple comparisons test. * Indicates significant difference (* *p* < 0.05, ** *p* < 0.01) between control and H_2_O_2_. # represents a significant difference (# *p* < 0.05, ## *p* < 0.01, ### *p* < 0.001) between H_2_O_2_ and compound-H_2_O_2_ groups. CTL, control.

The expression patterns obtained using RT-qPCR were highly consistent with those identified using transcriptome sequencing (Figure 6). Specifically, treatment with compound **C** significantly downregulated the expression of *S1pr1* compared with that in the H_2_O_2_ model group (*p* < 0.01). In contrast, the mRNA expression levels of *Adora2a*, *Adm*, *Tbxa2r*, and *Grin3b* were markedly increased following compound **C** treatment (*p* < 0.05).

Notably, *Adora2a* and *Tbxa2r* are key components of GPCR-mediated signaling, whereas *Grin3b* is involved in neuronal excitability and calcium homeostasis. The altered expression of these genes suggests that compound **C** may exert neuroprotective effects through coordinated regulation of GPCR signaling, cell adhesion-related processes, and calcium channel-associated pathways. Overall, the RT-qPCR results further verified the accuracy and reliability of the RNA-seq analysis and supported the involvement of GPCR-related signaling pathways in compound **C**-mediated neuroprotection.

### 2.7. Compound ***C*** Increased the Phosphorylation Level of PKA Cα at Thr197 Under H_2_O_2_-Induced Oxidative Stress Conditions, Suggesting Enhanced PKA Signaling Activity

Transcriptomic analyses were conducted on compound **C**. GPCR-related signaling pathways were significantly enriched. Additionally, the differential expression of several GPCR-associated genes, including *Adora2a* and *Tbxa2r*, was observed using RT-qPCR. To determine whether downstream PKA signaling was involved in the neuroprotective action of compound **C**, representative Western blot images and quantitative analysis of p-PKA Cα (Thr197) and total PKA Cα expression are shown in Figure 6F,G. The relative phosphorylation level of PKA Cα was calculated as the ratio of p-PKA Cα (Thr197) to total PKA Cα (*p* < 0.05), suggesting activation of PKA signaling under oxidative stress conditions. Notably, compound **C** treatment markedly attenuated H_2_O_2_-induced PKA phosphorylation (*p* < 0.05), whereas the total PKA protein level remained unchanged among the groups. These results indicate that compound **C** suppresses aberrant PKA activation induced by H_2_O_2_, implying that modulation of PKA signaling may contribute to its neuroprotective effects in C17.2 cells.

## 3. Discussion

All five compounds (**A**–**E**) isolated in the present study are previously reported known compounds rather than new chemical entities, and their structures were identified by comprehensive spectroscopic analyses and comparison with previously published spectroscopic data [16,17,18,19,20]. To the best of our knowledge, compound **C** was isolated from *S. commune* for the first time, thereby expanding the known phytochemical diversity of this medicinal mushroom. A critical prerequisite for evaluating the pharmacological potential of natural compounds is their safety profile [21]. In the present study, compound **C** at all tested concentrations exhibited negligible cytotoxicity toward C17.2 neural stem cells, supporting its suitability for further functional assays.

Importantly, compound **C** significantly alleviated H_2_O_2_-induced reductions in cell viability, suggesting a protective role against oxidative stress-induced cellular injury. Oxidative stress is a central pathological mechanism implicated in neuronal damage and neurodegenerative disorders, primarily through excessive ROS accumulation and subsequent macromolecular damage. Therefore, the observed protective effects of compound **C** suggest its potential involvement in maintaining cellular redox homeostasis under oxidative conditions.

H_2_O_2_ is widely used as an in vitro model to induce oxidative stress in neuronal cells [22]. Consistent with our findings, previous studies have demonstrated that H_2_O_2_ exposure significantly reduces neuronal viability through ROS overproduction and mitochondrial dysfunction [23]. Flavonoid compounds, such as quercetin and luteolin, alleviate H_2_O_2_-induced cytotoxicity in neuronal cells by restoring redox homeostasis and suppressing apoptotic signaling pathways [24]. These observations are consistent with the protective effects observed for compound **C**, suggesting that modulating oxidative stress remains a common mechanism underlying neuroprotection induced by natural products.

Oxidative stress can activate multiple intracellular signaling cascades, including kinase-dependent pathways that regulate cell survival and apoptosis [25,26]. Therefore, the protective effect of compound **C** may be associated with direct antioxidant properties as well as stress-responsive signaling network modulation. The non-linear concentration-response pattern observed for compound **C** may represent a hormetic response, a phenomenon frequently reported for bioactive compounds, in which low concentrations activate adaptive protective pathways while higher concentrations may attenuate these beneficial effects.

To elucidate the molecular basis of compound **C**-mediated neuroprotection, transcriptomic analysis was performed. GPCR-related signaling pathways, including neuroactive ligand–receptor interaction and downstream intracellular signaling cascades, were significantly involved in compound **C**-mediated neuroprotection. GPCR-mediated signaling pathways have been increasingly recognized as key regulators of neuronal survival and stress adaptation [27,28]. The enrichment of GPCR-associated pathways suggests that compound **C** may exert its neuroprotective effects through modulation of neurotransmitter receptor signaling networks rather than acting solely as a direct antioxidant. This interpretation is consistent with the emerging view that natural products often exert pleiotropic effects by targeting multiple signaling pathways rather than a single molecular target [10].

The qPCR results were highly consistent with transcriptomic data, confirming the reliability of the sequencing analysis. Among these genes, *Adora2a* encodes the adenosine A2A receptor, a well-characterized GPCR involved in neuromodulation and neuroprotection [29]. Adenosine signaling regulates neuronal survival under stress conditions by modulating excitability and inflammatory responses [30]. Similarly, S1PR1 signaling regulates neuronal inflammation and cell survival in stress-related models [31]. Adrenomedullin (*Adm*) participates in vasodilatory and stress-responsive signaling and may exert protective effects under cellular stress conditions [32,33]. *Tbxa2r*, a GPCR involved in thromboxane signaling, may contribute to the regulation of intracellular calcium and inflammatory responses under oxidative stress conditions [34]. *Grin3b*, a subunit of *NMDA*-type glutamate receptors, is implicated in synaptic transmission and calcium-mediated neuronal excitotoxicity, which may be indirectly associated with oxidative stress regulation [35]. The coordinated regulation of these GPCR-related genes suggests that compound **C** may restore signaling balance within neuroactive ligand–receptor networks, thereby contributing to cellular resilience against oxidative stress.

Given the enrichment of GPCR signaling pathways, we further investigated downstream signaling events, particularly the PKA pathway, which is a canonical effector of GPCR-mediated cAMP signaling. PKA is a key serine/threonine kinase activated by intracellular cAMP and plays a central role in regulating neuronal survival, synaptic plasticity, and metabolic adaptation [36]. Dysregulated PKA activity is associated with cellular stress responses and neuronal dysfunction. In the present study, H_2_O_2_ exposure significantly increased the phosphorylated PKA level without affecting total PKA expression, indicating PKA signaling activation under oxidative stress conditions. Notably, compound **C** significantly attenuated H_2_O_2_-induced PKA phosphorylation. Similar observations were reported in oxidative stress models, where aberrant activation of cAMP/PKA signaling contributes to cellular injury and its modulation exerts protective effects [37]. These findings suggest that regulation of PKA phosphorylation may be involved in cellular responses to oxidative stress.

Based on transcriptomic and biochemical analyses, a working model for the neuroprotective effects of compound **C** can be proposed. Compound **C** may modulate GPCR-associated signaling networks and suppress aberrant PKA phosphorylation induced by oxidative stress, thereby contributing to improved C17.2 cell survival. GPCR-mediated cAMP/PKA signaling is a critical pathway linking extracellular stimuli to intracellular responses in neuronal cells. The involvement of this pathway is supported by previous studies demonstrating its role in neuronal survival and mitochondrial regulation [38,39]. Therefore, modulation of this signaling axis may be a key mechanism underlying the observed neuroprotective effects.

A limitation of this study is that a positive cytotoxicity control was not included; therefore, the results only indicate that compound **C** did not significantly reduce C17.2 cell viability under the tested conditions. Although increased phosphorylation of PKA Cα at Thr197 suggests enhanced PKA signaling activity, additional investigations, including direct measurement of intracellular cAMP levels, CREB phosphorylation analysis, and pharmacological modulation of PKA activity, are required to further confirm the involvement of the cAMP/PKA signaling pathway. The use of an immortalized murine C17.2 neural stem cell line also limits the generalizability of the findings, and future studies using human-derived neural cells and in vivo models are needed. Moreover, a broader concentration range and additional oxidative stress and cell death-related assays would help further characterize the dose–response relationship and protective effects of compound **C**.

## 4. Materials and Methods

### 4.1. General Information

HPLC analysis was performed with an Agilent 1260 HPLC system (Agilent Technologies, Santa Clara, CA, USA) equipped with a G1311B quaternary pump and G1315D diode array detector (DAD) using a ZORBAX SB-C18 column (4.6 × 250 mm, 5 μm). Nuclear magnetic resonance (NMR) spectra were recorded on an AVANCE NEO 600 spectrometer (Bruker, Billerica, MA, USA) using tetramethylsilane (TMS) as the internal standard. High-resolution electrospray ionization mass spectrometry (HR-ESI-MS) data were acquired using a Triple TOF 5600 system (AB SCIEX, Framingham, MA, USA) to determine the molecular weights of the isolated compounds.

### 4.2. Fungal Material

The fruiting bodies of *S*. *commune* were provided by Dr. Shengjie Li, Institute of Mycology, Nanjing Xiaozhuang University (Nanjing, China) and were identified as *S. commune* based on their morphological and taxonomic characteristics. The materials were pulverized, sieved through a 100-mesh screen, and freeze-dried to obtain the final powdered sample.

### 4.3. Extraction, Bioactivity-Guided Isolation and Structural Identification

#### 4.3.1. Preparation of Crude Extract

The dried fruiting bodies of *S. commune* (20 kg) were pulverized into powder and extracted three times with 90% methanol (material-to-solvent ratio, 1:2, *m*/*v*) at room temperature. The combined extracts were filtered and concentrated under reduced pressure using a rotary evaporator to yield 32.6 g of crude methanol extract. Methanol (analytical grade) is produced by Shanghai Titan Scientific Co., Ltd., Shanghai, China.

#### 4.3.2. MPLC Fractionation

The crude methanol extract was subjected to reversed-phase C18 medium-pressure liquid chromatography (MPLC) (Orienda Instrument Co., Ltd., Beijing, China). Gradient elution was performed using water (Wahaha Group Co., Ltd., Hangzhou, China) and methanol (HPLC grade, Sigma-Aldrich Trading Co., Ltd., Shanghai, China) as the mobile phases. Based on the chromatographic profiles from HPLC, sixteen fractions (Fr.1–Fr.16) were collected and concentrated under reduced pressure for subsequent bioactivity screening.

#### 4.3.3. Bioactivity-Guided Screening of Fractions

The neuroprotective activities of the obtained fractions were evaluated using an H_2_O_2_-induced oxidative stress model in C17.2 neural stem cells. Cells were pretreated with different fractions prior to oxidative injury induction. Cell viability was determined using the Cell Counting Kit-8 (CCK-8) assay, and fractions exhibiting significant protective effects were selected for further purification.

#### 4.3.4. Semi-Preparative HPLC Purification

The active fractions identified through bioactivity-guided screening were further purified by semi-preparative HPLC using an Agilent 1260 HPLC system (Agilent Technologies, Santa Clara, CA, USA) equipped with a ZORBAX SB-C18 column (4.6 × 250 mm, 5 μm). Isocratic elution was performed using acetonitrile and 0.1% formic acid in water at a flow rate of 2.0 mL/min. The injection volume was 20 μL. The elution conditions for compounds **A**–**E** were as follows: acetonitrile/0.1% formic acid in water, 33:67 (*v*/*v*) for compounds **A** and **B**; 35:65 (*v*/*v*) for compound **C**; 22:78 (*v*/*v*) for compound **D**; and 60:40 (*v*/*v*) for compound **E**. Detection was performed at 280 nm for compounds **A**, **B**, and **D** and at 254 nm for compounds **C** and **E**. Finally, five compounds, designated as compounds **A**–**E**, were isolated. Multiple purification procedures were conducted to obtain pure compounds. Finally, five compounds, designated as compounds **A**–**E**, were isolated (**A**: 15.6 mg; **B**: 6.8 mg; **C**: 13.6 mg; **D**: 3 mg; **E**: 20.9 mg).

#### 4.3.5. Structural Elucidation of Compounds **A**–**E**

The structures of compounds **A**–**E** were elucidated by comprehensive spectroscopic analyses, including HR-ESI-MS, 1H NMR, 13C NMR, distortionless enhancement by polarization transfer (DEPT), correlation spectroscopy (COSY), heteronuclear single quantum coherence (HSQC), and heteronuclear multiple bond correlation (HMBC) experiments. The obtained spectroscopic data were compared with previously reported literature data for structural identification.

### 4.4. Cell Culture

The C17.2 mouse neural stem cell line was purchased from Yimo Biotechnology Co., Ltd. (Xiamen, China; catalog no. IM-M072). C17.2 neural stem cells were cultured in Dulbecco’s modified Eagle’s medium (DMEM) supplemented with 10% fetal bovine serum (Shenghang Biotechnology Co., Ltd., Nanjing, China) and 1% penicillin–streptomycin (GIBCO, Grand Island, NY, USA). Cells were maintained in a humidified incubator containing 5% CO_2_ at 37 °C. The culture medium was replaced every 2–3 days, and cells in the logarithmic growth phase were used for all experiments.

### 4.5. Cell Viability

#### 4.5.1. Cytotoxicity Evaluation of Compounds **A**–**E**

The cytotoxicities of compounds **A**–**E** toward C17.2 cells were evaluated using the CCK-8 assay. Cells were seeded into 96-well plates and incubated overnight. Subsequently, cells were treated with different concentrations of compounds (0.1, 1, 10, 100 μg/mL) **A**–**E** for 24 h. After treatment, CCK-8 solution was added to each well and incubated for an additional period according to the manufacturer’s instructions. The absorbance was measured at 450 nm using a microplate reader (Boten Instrument Co., Ltd., Shanghai, China).

#### 4.5.2. Neuroprotective Effects Against H_2_O_2_-Induced Oxidative Stress

Compound **C** was dissolved in dimethyl sulfoxide (DMSO) and diluted with complete culture medium before treatment. The final concentration of DMSO was maintained at the same level in all treatment groups, and the vehicle control group was included in parallel experiments. Based on the dose–response analysis, 1 μg/mL compound **C**, which showed the strongest protective effect against H_2_O_2_-induced cytotoxicity, was selected for subsequent mechanistic investigations. H_2_O_2_ was prepared by diluting a 3% H_2_O_2_ solution with culture medium to obtain a final concentration of 600 μM, which was is produced by Lircon Disinfection Technology Co., Ltd. Briefly, cells were pretreated with compounds **A**–**E** at different concentrations for 24 h, followed by exposure to 600 μM H_2_O_2_ for 3 h. Cell viability was then determined using the CCK-8 assay. The protective effects of the compounds against oxidative stress-induced cell injury were evaluated based on cell survival rates.

### 4.6. Oxidative Stress Evaluation

#### 4.6.1. Intracellular ROS Measurement

Intracellular reactive oxygen species (ROS) levels were measured using the fluorescent probe DCFH-DA which was produced by Lircon Disinfection Technology Co., Ltd., Dezhou, China. C17.2 cells with a density of 3 × 10^4^ cells/mL were inoculated into 96-well plates at a rate of 100 μL per well and cultured in an incubator for about 24 h. After treatment, cells were incubated with DCFH-DA working solution at 37 °C in the dark. Subsequently, cells were washed with phosphate-buffered saline (PBS) to remove excess probe. Fluorescence images were captured using an inverted microscope (Olympus Corporation, Tokyo, Japan), and fluorescence intensity was quantified using ImageJ software.

#### 4.6.2. Determination of MDA Content

The intracellular malondialdehyde (MDA) content was determined using a commercial assay kit according to the manufacturer’s instructions. C17.2 cells with a density of 1 × 10^5^ cells/mL were inoculated into 6-well plates at a rate of 2 mL per well and cultured in an incubator for about 24 h. After treatment, cells were collected and lysed, and the supernatants were used for MDA measurement. The absorbance was measured using a microplate reader, and the results were normalized to protein concentration which was determined by the Bicinchoninic Acid Assay (BCA).

#### 4.6.3. Determination of SOD Activity

Superoxide dismutase (SOD) activity was determined using a commercial assay kit following the manufacturer’s protocol. C17.2 cells with a density of 1 × 10^5^ cells/mL were inoculated into 6-well plates at a rate of 2 mL per well and cultured in an incubator for about 24 h. Cell lysates were prepared after treatment, and SOD activity was measured spectrophotometrically. The enzyme activity was normalized to total protein concentration which was determined by BCA.

#### 4.6.4. Determination of CAT Activity

Catalase (CAT) activity was measured using a commercial assay kit according to the manufacturer’s instructions. Briefly, C17.2 cells with a density of 1 × 10^5^ cells/mL were inoculated into 6-well plates at a rate of 2 mL per well and cultured in an incubator for about 24 h. Treated cells were lysed, and the decomposition rate of hydrogen peroxide was monitored spectrophotometrically. CAT activity was normalized to protein concentration.

### 4.7. Transcriptome Experiment and Analysis

#### 4.7.1. RNA Extraction and Library Construction

Total RNA was extracted from treated C17.2 cells using a commercial RNA extraction kit according to the manufacturer’s instructions. RNA sequencing was performed by Shanghai OE Biotech Co., Ltd., Shanghai, China. Three biological replicates were analyzed for each experimental group. Differentially expressed genes (DEGs) were identified based on comparisons among different experimental groups. Genes with |log_2_ fold change| ≥ 1 and adjusted *p*-value < 0.05 were considered significantly differentially expressed.

#### 4.7.2. RNA Sequencing

To investigate the molecular mechanisms underlying the neuroprotective effects of compound **C**, transcriptomic analysis was performed on C17.2 cells from the control group, H_2_O_2_-induced model group, and compound **C**-treated group using RNA sequencing (RNA-seq). Total RNA was extracted using trizol reagent according to the manufacturer’s instructions. RNA quality and integrity were evaluated prior to library construction.

Messenger RNA (mRNA) was enriched from total RNA using oligo(dT)-attached magnetic beads and subsequently fragmented into short fragments. First-strand and second-strand complementary DNA (cDNA) were then synthesized and used for sequencing library preparation. The constructed libraries were sequenced on an Illumina sequencing platform by OE Biotechnology Co., Ltd. (Shanghai, China).

After quality control and filtering of raw sequencing data, clean reads were aligned to the reference genome for downstream analysis. Differentially expressed genes (DEGs) among groups were identified using DESeq2 software (version 1.38.3).

#### 4.7.3. GO, KEGG and WikiPathways Enrichment Analysis

Principal component analysis (PCA), volcano plots, and hierarchical clustering heatmaps were generated to evaluate sample distribution and visualize DEG profiles. Functional enrichment analyses, including Gene Ontology (GO) annotation, Kyoto Encyclopedia of Genes and Genomes (KEGG) and WikiPathways analysis, were subsequently carried out to explore the biological functions and signaling pathways associated with the identified DEGs.

### 4.8. Quantitative Real-Time PCR

#### 4.8.1. RNA Isolation and Reverse Transcription

Total RNA was extracted from treated C17.2 cells using a commercial RNA extraction kit according to the manufacturer’s instructions. The concentration and purity of the isolated RNA were determined spectrophotometrically. Subsequently, complementary DNA (cDNA) was synthesized from total RNA using a reverse transcription kit following the manufacturer’s protocol. The obtained cDNA samples were stored at −20 °C until further analysis.

#### 4.8.2. Quantitative PCR

Quantitative real-time PCR (qRT-PCR) was performed using a real-time PCR system with a SYBR Green-based detection method. The amplification reaction was carried out in a final reaction volume according to the manufacturer’s instructions. The thermal cycling conditions consisted of an initial denaturation step followed by repeated cycles of denaturation, annealing, and extension. Each sample was analyzed in triplicate to ensure reproducibility.

#### 4.8.3. Relative Gene Expression Analysis

The relative mRNA expression levels of target genes were normalized to β-actin expression as an internal control (Table 1). Relative gene expression was calculated using the comparative Ct method: 2^−ΔΔCt^. The results were expressed as fold changes relative to the control group.

### 4.9. Western Blotting

For Western blot analysis, cells were lysed in ice-cold RIPA buffer supplemented with protease and phosphatase inhibitor cocktails. The lysates were subsequently centrifuged at 12,000 rpm for 10 min at 4 °C using a Sorvall Legend Micro 21R centrifuge (Thermo Fisher Scientific, Waltham, MA, USA). Protein concentrations were determined using a bicinchoninic acid (BCA) protein assay kit (E112-02, Vazyme, Nanjing, China) with bovine serum albumin as the calibration standard.

Equivalent amounts of protein from each sample were resolved by SDS–PAGE and electrotransferred onto polyvinylidene fluoride (PVDF) membranes (ISEQ00010, Millipore, Bedford, MA, USA). Following transfer, the membranes were blocked with 5% non-fat milk for 2 h at room temperature and subsequently incubated with primary antibodies overnight at 4 °C. After washing, the membranes were incubated with HRP-conjugated secondary antibodies for 1 h at room temperature.

The primary antibodies used in this study included rabbit anti-phospho-PKA Cα (5661, Cell Signaling Technology, Danvers, MA, USA), rabbit anti-PKA Cα (5842, Cell Signaling Technology, Danvers, MA, USA), and mouse anti-GAPDH (60004-I-Ig, Proteintech, Wuhan, China). Immunoreactive bands were detected using a Tanon-5200 chemiluminescence imaging system (180-501, Tanon Science & Technology Co., Ltd., Shanghai, China). Band intensities were quantified with ImageJ software (Version win64, National Institutes of Health, Bethesda, MD, USA), normalized to the corresponding internal control, and expressed as fold changes relative to the control group. The phosphorylation status of PKA Cα was evaluated using an anti-phospho-PKA Cα (Thr197) antibody. The expression level of phosphorylated PKA Cα was normalized to total PKA Cα protein levels.

### 4.10. Statistics Analysis

All experiments were performed with at least three independent biological replicates. Statistical analyses were conducted using GraphPad Prism version 8.0.1 (GraphPad Software 8.0.1, San Diego, CA, USA). Data are presented as mean ± standard deviation (SD) from three independent biological experiments (*n* = 3). For comparisons between two groups, an unpaired Student’s *t*-test was used. For comparisons involving more than two groups, one-way analysis of variance (ANOVA) followed by Tukey’s multiple comparisons test was performed. A *p*-value < 0.05 was considered statistically significant. Sample sizes were determined based on our previous experience and on similar studies reported in the literature. Quantitative data are presented as the mean ± SEM. Detailed information regarding sample size, statistical methods, and significance levels is provided in the corresponding figure legends.

## 5. Conclusions

In this study, five compounds were isolated from *S. commune*, among which Compound **C** exhibited protective activity against H_2_O_2_-induced oxidative stress injury in C17.2 neural stem cells. Compound **C** modulated genes associated with GPCR-related signaling pathways, *Adora2a*, *S1pr1*, *Adm*, *Tbxa2r*, and *Grin3b*. Additionally, compound **C** significantly reduced H_2_O_2_-induced PKA phosphorylation without affecting total protein levels, suggesting regulation of the cAMP/PKA signaling pathway. These findings suggest that compound **C** may exert neuroprotective effects through modulation of GPCR-associated signaling networks, with the cAMP/PKA pathway potentially involved in this process. Overall, this study highlights *S. commune* as a promising source of bioactive compounds and provides new insights into the molecular mechanisms potentially underlying its protective effects against oxidative stress-induced cellular injury.

## Figures and Tables

**Table 1:** Sequence list of gene primers.

Genes	Forward Primer (5′ to 3′)	Reverse Primer (5′ to 3′)
*Actin*	TGTCCACCTTCCAGCAG	CGCAGCTCAGTAACAGTCC
*Tbxa2r*	TCGGGCTCATATTCGCACTC	CCCACGAGCTGAACCATCAT
*Adm*	CATCCAGCAGCTACCCTACG	GTGTTCTGCTCGTCCAGGAA
*S1pr1*	TCTGGCTGTGCTGAACTCAG	GCGGCTAAATTCCATGCCTG
*Adora2a*	ACTGCAGAAGGAAGTCCACG	ACGTGGAGCAGAAGAAGGTG
*Grin3b*	GGCTCCAAAGTTTGTGCTGG	TGCTGCCTCTAGGACCTCAT

## Data Availability

Data are contained within the article and Supplementary Materials.

## Supplementary Materials

The following supporting information can be downloaded at: https://www.mdpi.com/article/10.3390/ijms27177974/s1.

## Abbreviations

The following abbreviations are used in this manuscript:

cAMP	Cyclic adenosine monophosphate
CAT	Catalase
CTL	Control
DEG	Differentially expressed gene
GO	Gene Ontology
GPCR	G protein coupled receptor
KEGG	Kyoto Encyclopedia of Genes and Genomes
MDA	Malondialdehyde
PCA	Principal component analysis
PKA	Protein kinase A
RNA-seq	RNA sequencing
ROS	Reactive oxygen species
