# Supplementary material for: A Sesquiterpenoid from Schizophyllum commune Protects C17.2 Neural Stem Cells Against Oxidative Stress Through Modulation of GPCR-Associated Signaling Pathways
Source: Int J Mol Sci. 2026 Sep 7;27(17):7974. doi: 10.3390/ijms27177974 (PMC13565997; doi:10.3390/ijms27177974)
Supplement: Supplementary file 1 [file ijms-27-07974-s001.zip › ijms-4470481-supplementary.pdf]

Supplrmentary Materials include Supplrmentary Figures S1-S19

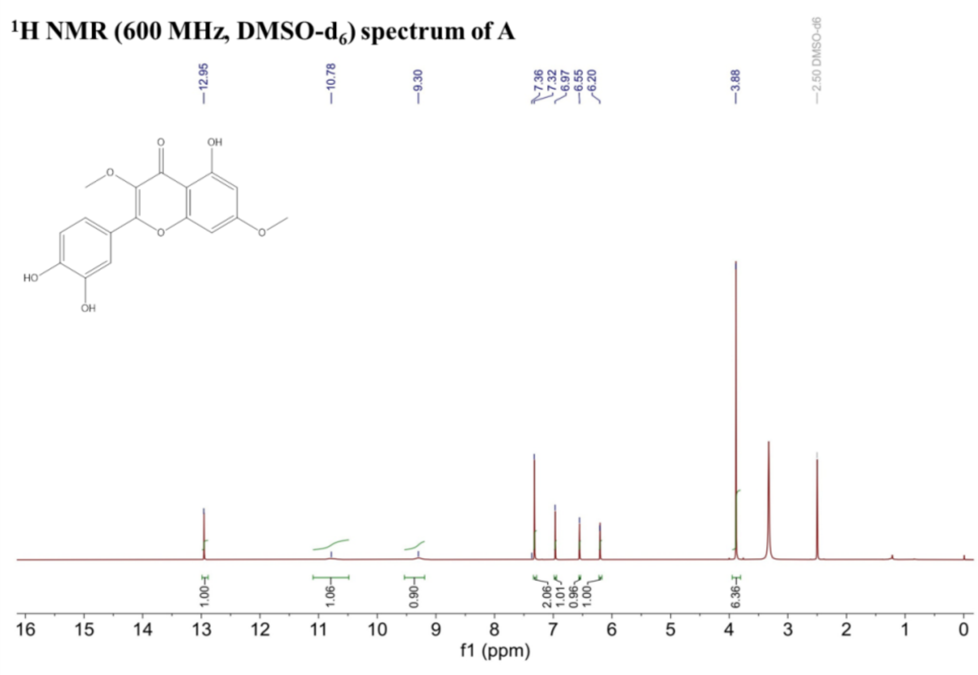

Supplementary Figure S1. ^{1}H NMR spectrum of compound A (600 MHz, DMSO-d$_6$)

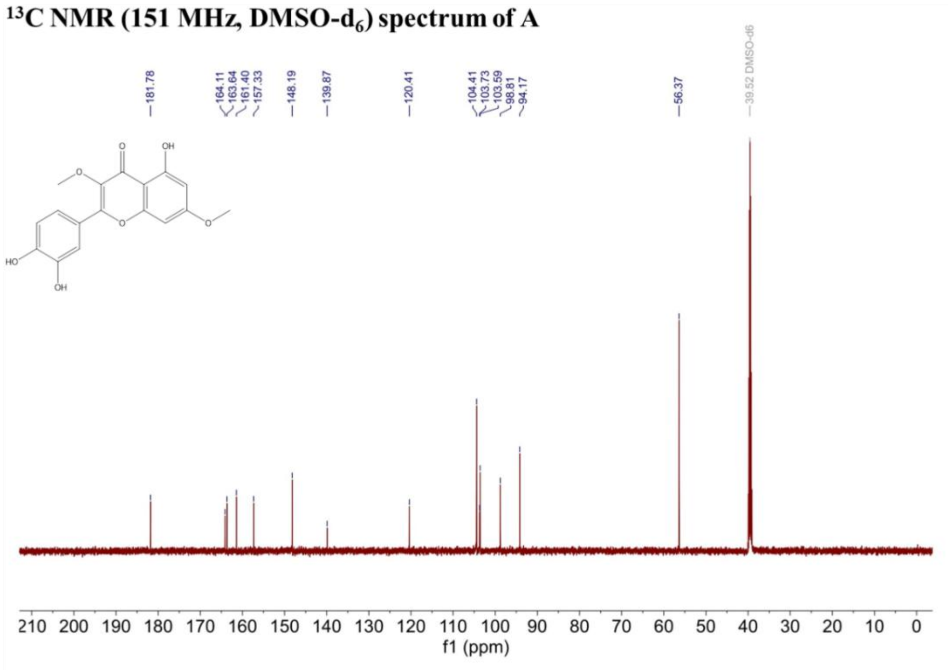

Supplementary Figure S2. ^{13}C NMR spectrum of compound A (151 MHz, DMSO-d$_6$)

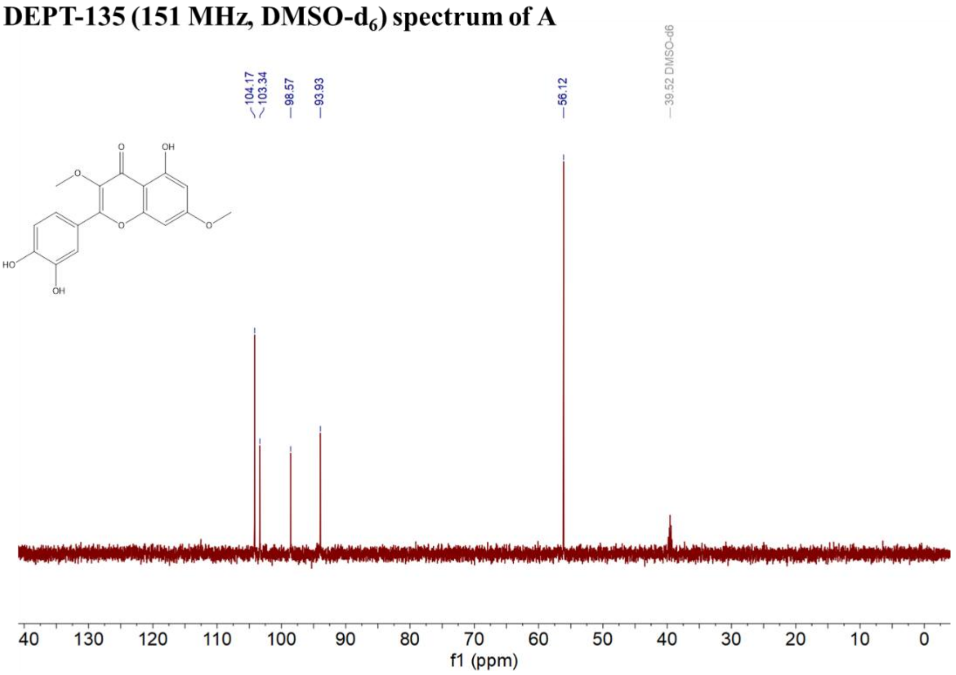

Supplementary Figure S3. DEPT-135 spectrum of compound A (151 MHz, DMSO-d_6)

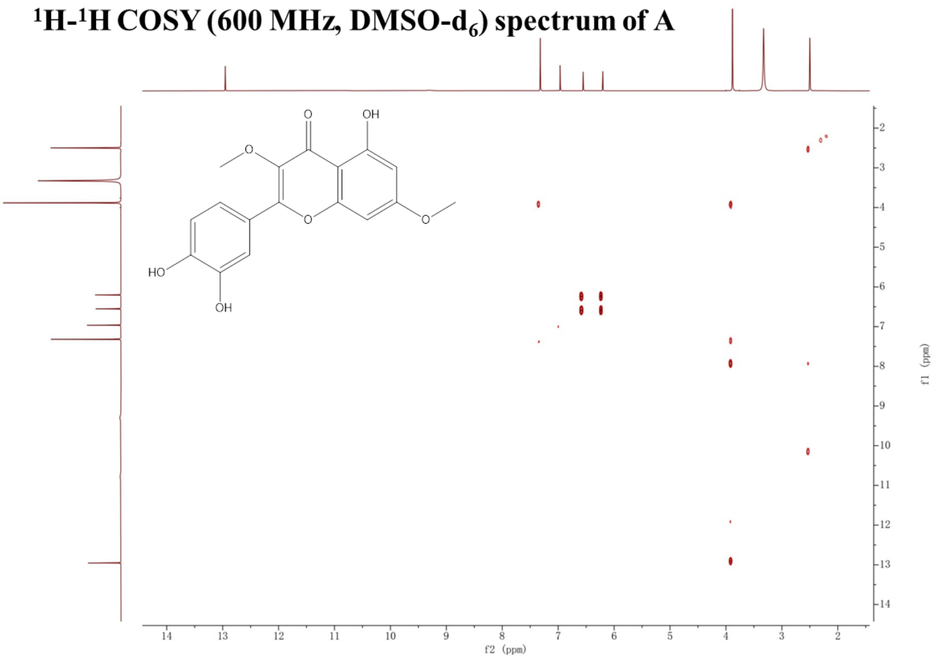

Supplementary Figure S4. ^1H-^1H COSY spectrum of compound A (600 MHz, DMSO- d_6)

HSQC (600 MHz, DMSO-d_6) spectrum of A

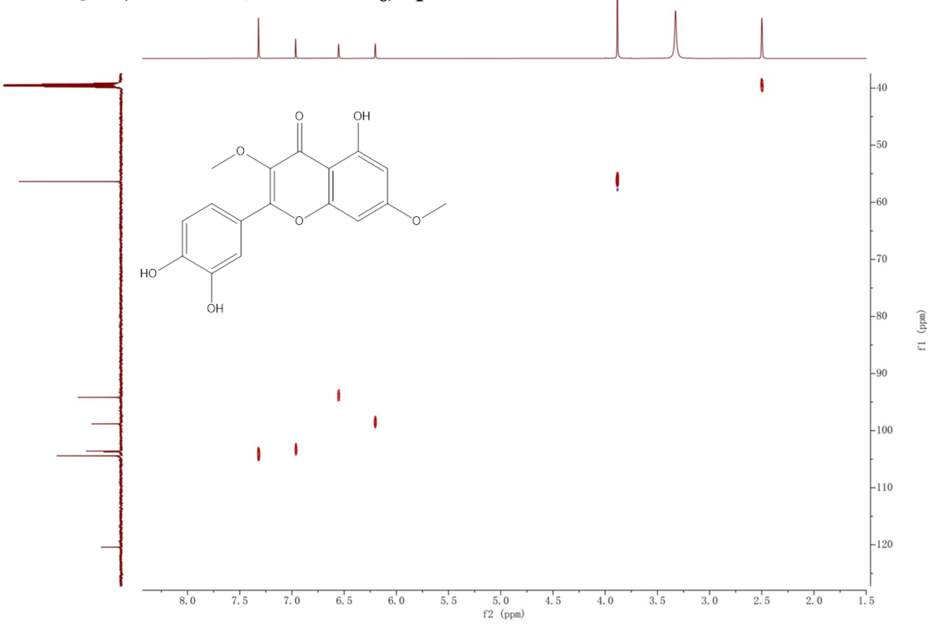

Supplementary Figure S5. HSQC spectrum of compound A (600 MHz, DMSO- d_6)

HMBC (600 MHz, DMSO-d_6) spectrum of A

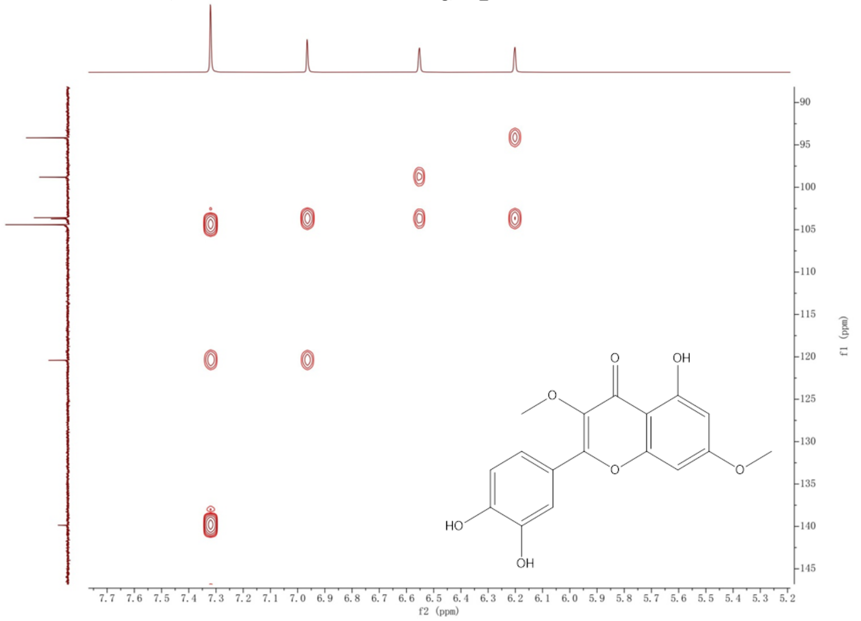

Supplementary Figure S6. HMBC spectrum of compound A (600 MHz, DMSO- d_6)

^{1}H NMR (600 MHz, CD_3OD) spectrum of B

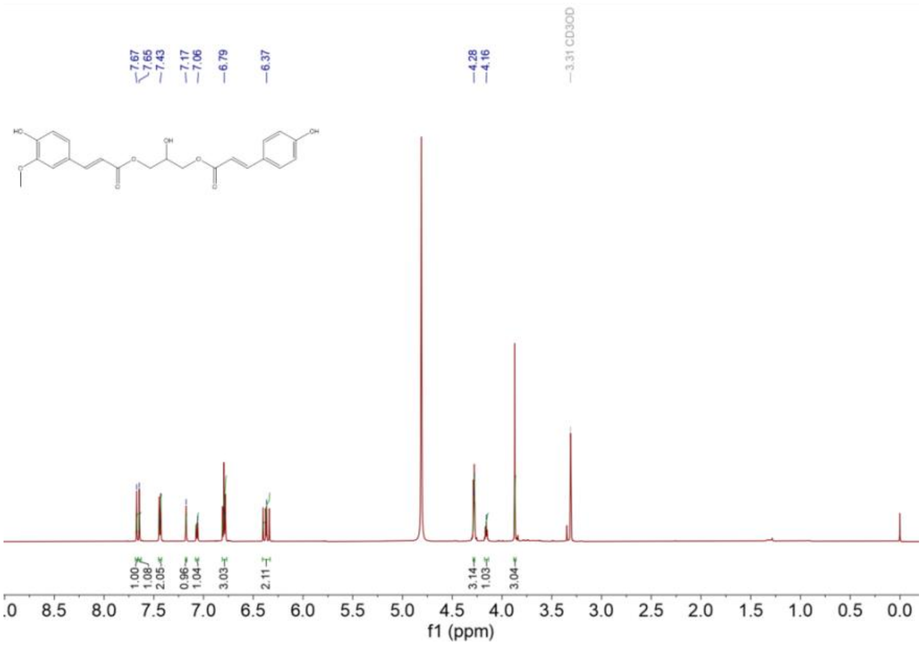

Supplementary Figure S7. ^{1}H NMR spectrum of compound B (600 MHz, MeOD)

^{13}C NMR (151 MHz, CD_3OD) spectrum of B

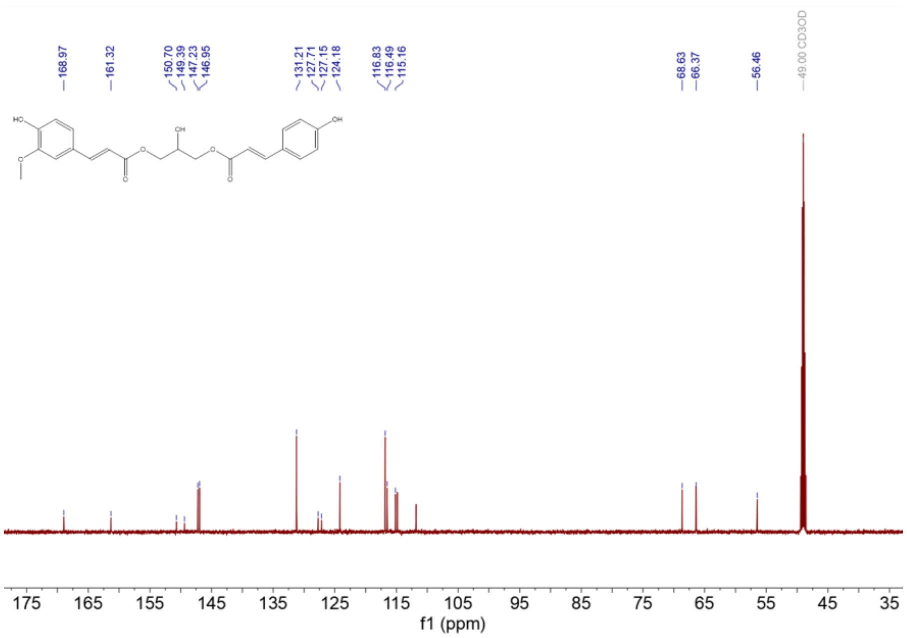

Supplementary Figure S8. ^{13}C NMR spectrum of compound B (151 MHz, DMSO-d_6)

^{1}H NMR (600 MHz, CD_3OD) spectrum of C

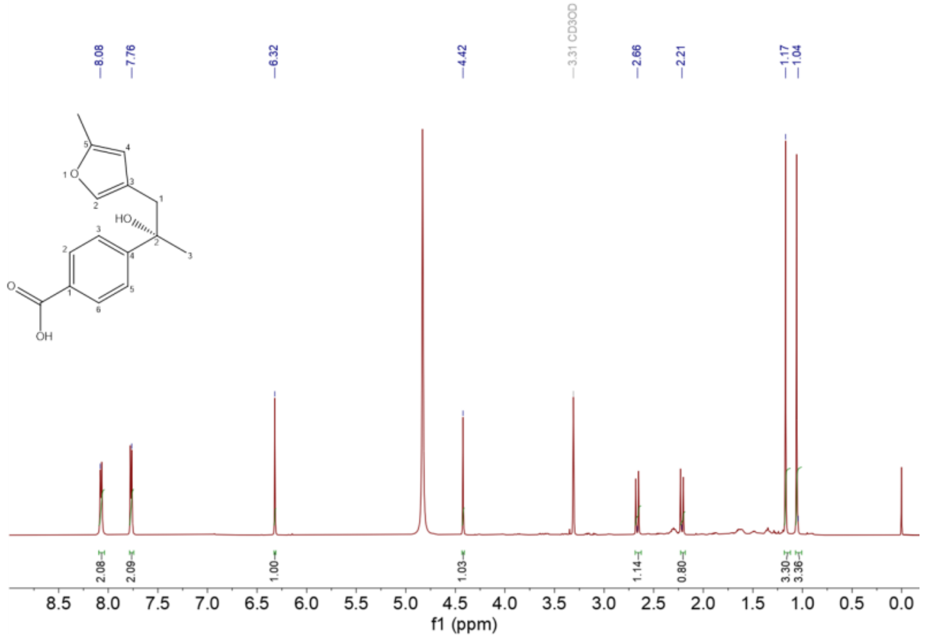

Supplementary Figure S9. ^{1}H NMR spectrum of compound C (600 MHz, MeOD)

^{13}C NMR (151 MHz, CD_3OD) spectrum of C

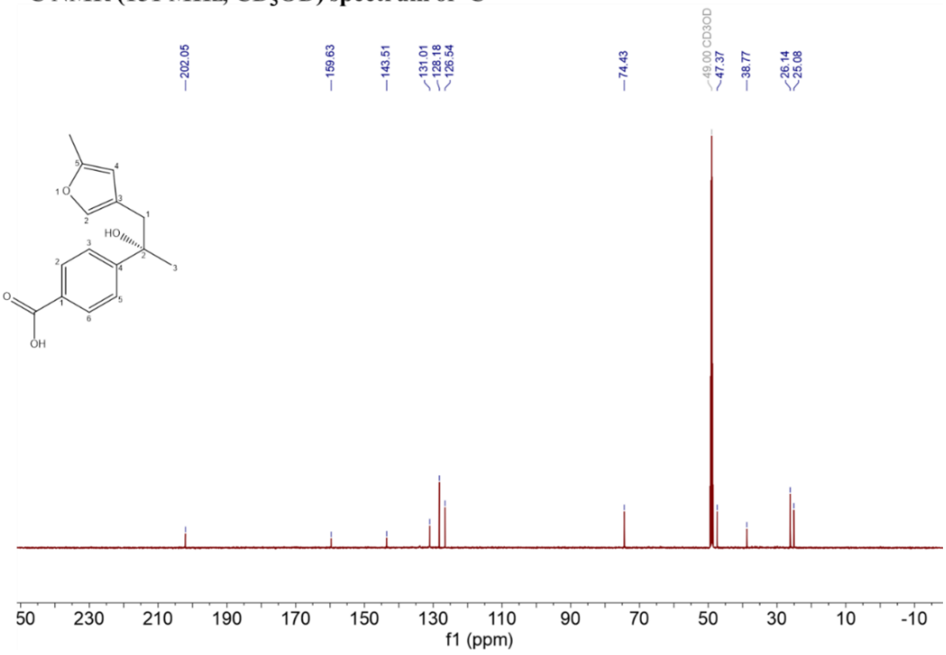

Supplementary Figure S10. ^{13}C NMR spectrum of compound C (151 MHz, MeOD)

^1H NMR (600 MHz, CD_3OD) spectrum of D

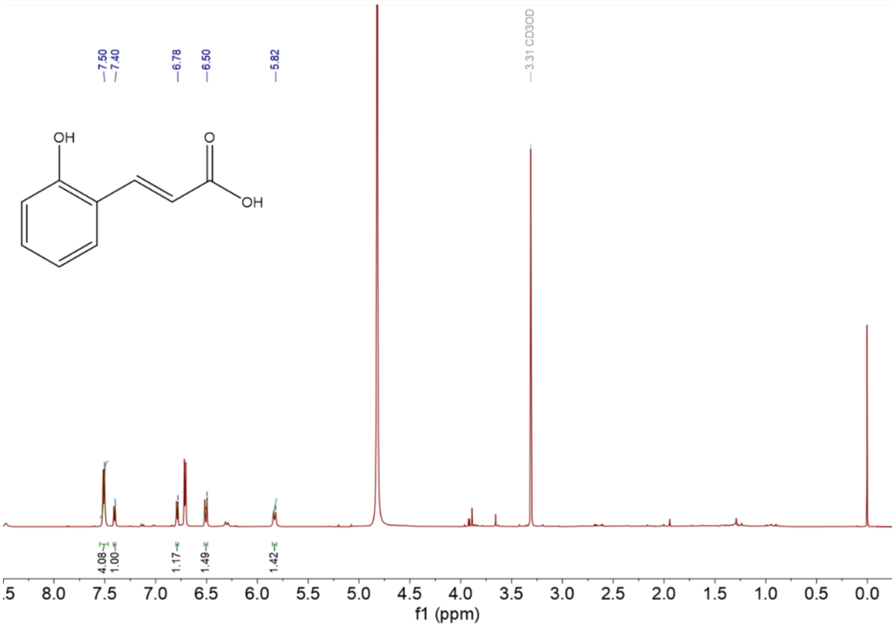

Supplementary Figure S11. ^1H NMR spectrum of compound D (600 MHz, MeOD)

^{13}C NMR (151 MHz, CD_3OD) spectrum of D

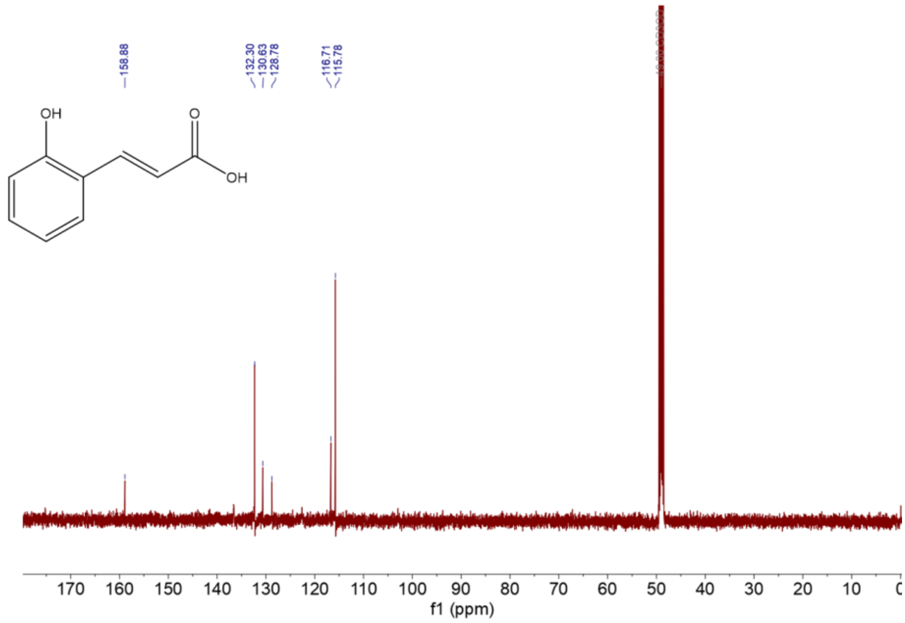

Supplementary Figure S12. ^{13}C NMR spectrum of compound D (151 MHz, MeOD)

^{1}H NMR (600 MHz, CD_3OD) spectrum of E

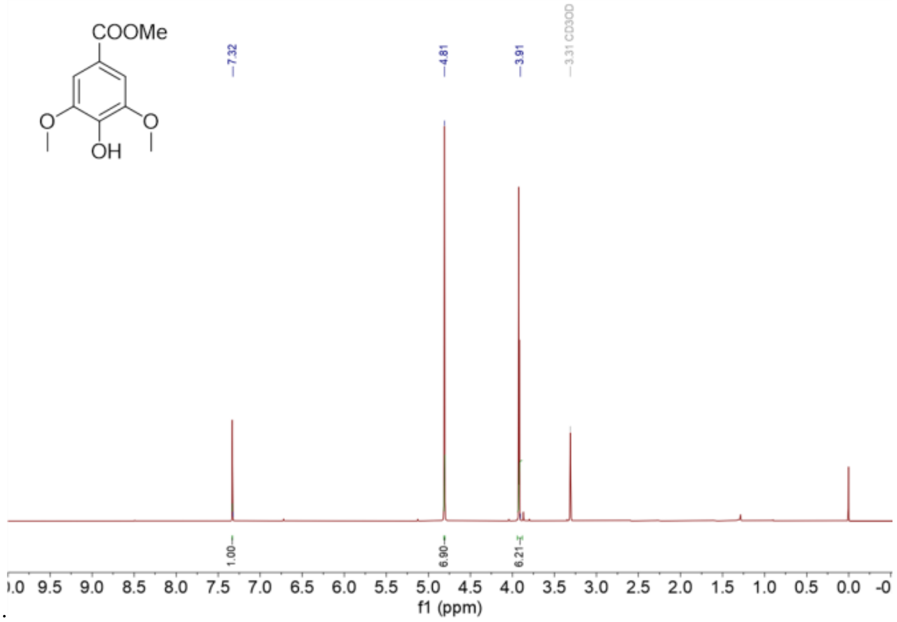

Supplementary Figure S13. ^{1}H NMR spectrum of compound E (600 MHz, MeOD)

^{13}C NMR (151 MHz, CD_3OD) spectrum of E

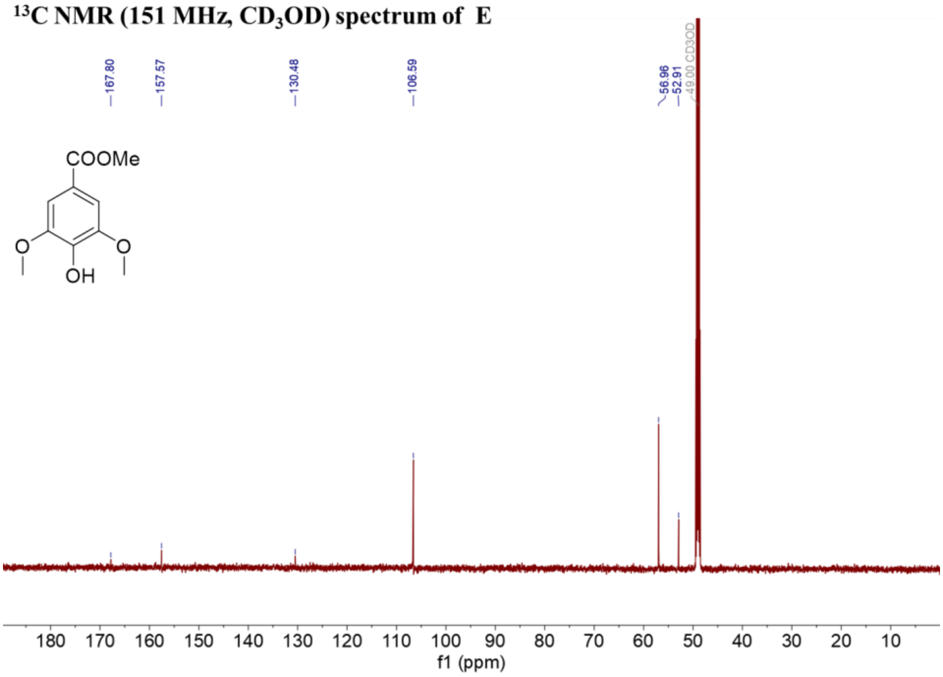

Supplementary Figure S14. ^{13}C NMR spectrum of compound E (151 MHz, MeOD)

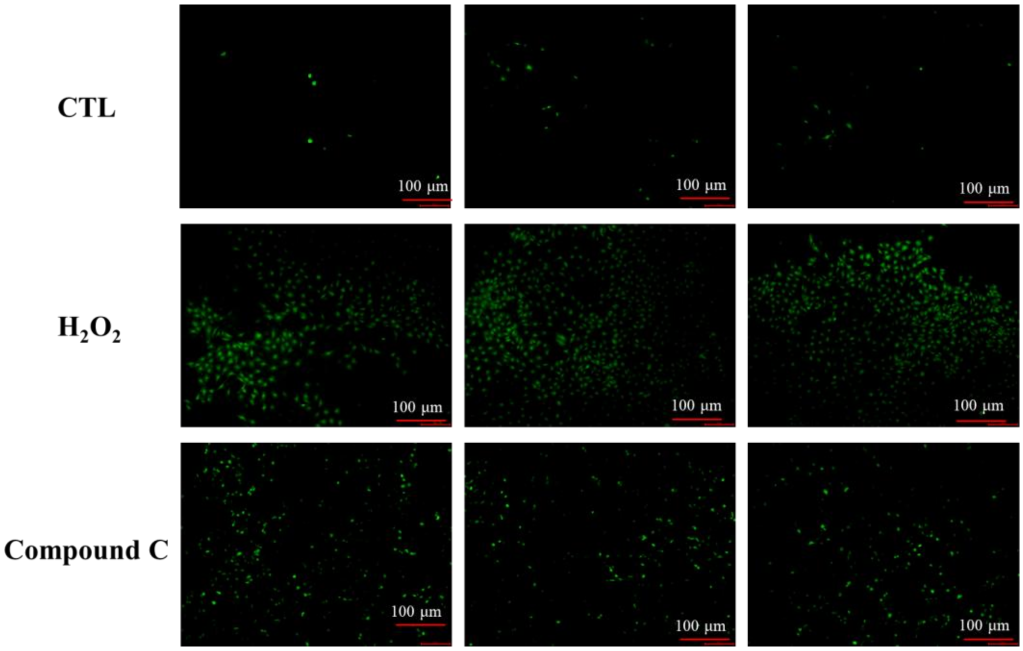

Supplementary Figure S15. Raw fluorescence images showing intracellular ROS levels in C17.2 neural stem cells. Intracellular reactive oxygen species (ROS) were detected using the DCFH-DA fluorescent probe. Representative fluorescence images from the control (CTL), H_2O_2-treated, and compound C + H_2O_2-treated groups are shown. The images were obtained directly from fluorescence microscopy without image processing. Scale bar: 100 μm.

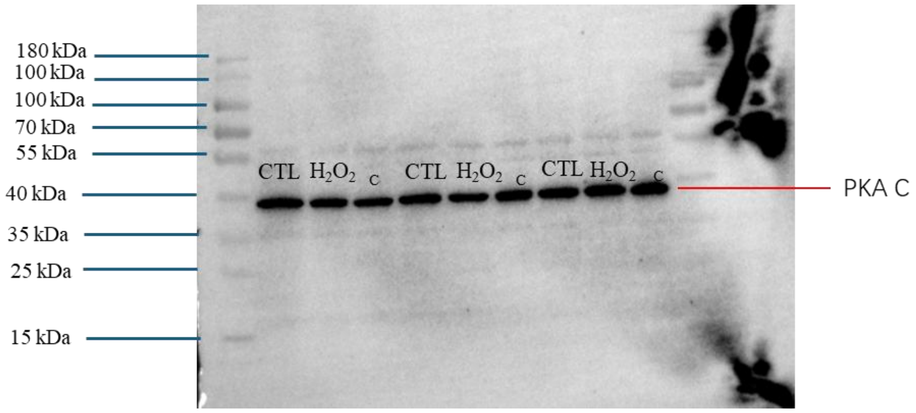

Supplementary Figure S16. Original uncropped Western blot images of total PKA C in C17.2 neural stem cells. Full-length blot images are shown without cropping or modification. Molecular weight markers are indicated on the left.

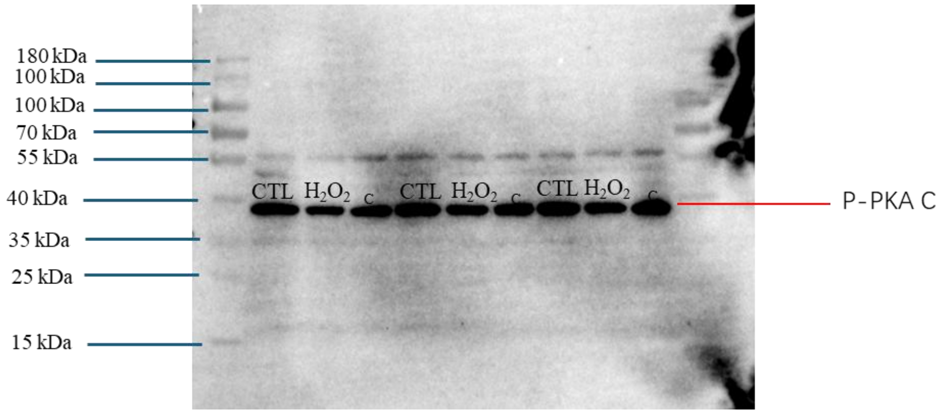

Supplementary Figure S17. Original uncropped Western blot images of phosphorylated PKA C (p-PKA C) in C17.2 neural stem cells. Full-length blot images are shown without cropping or modification. Molecular weight markers are indicated on the left.

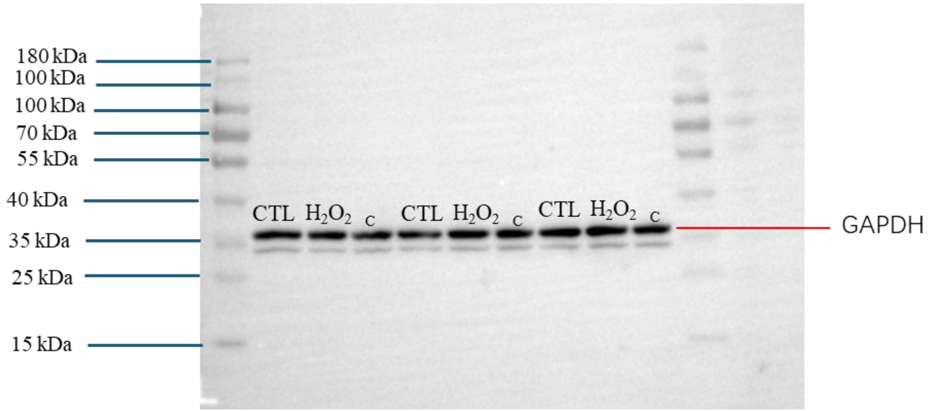

Supplementary Figure S18. Original uncropped Western blot images of GAPDH in C17.2 neural stem cells. Full-length blot images are shown without cropping or modification. Molecular weight markers are indicated on the left.

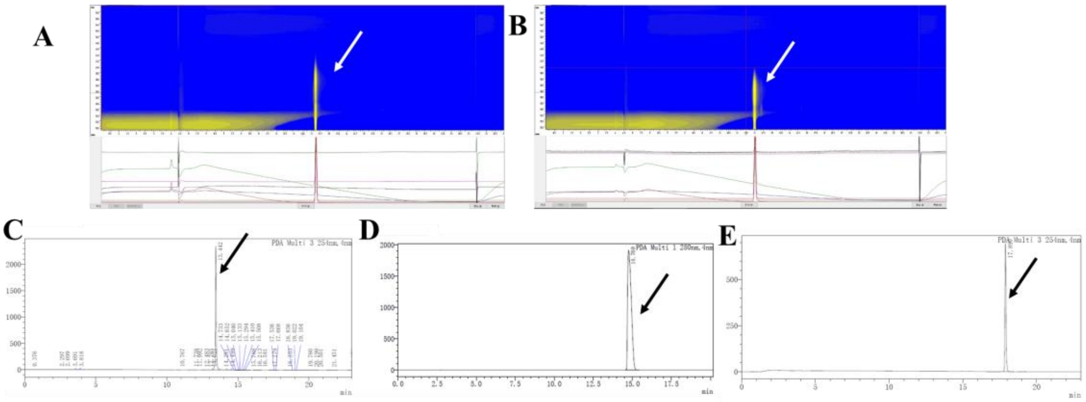

Figure S19. Analytical HPLC chromatograms of compounds **A-E**. (A) Compound A, tR = 12.2 min, detected at 280 nm; (B) Compound B, tR = 11.9 min, detected at 280 nm; (C) Compound C, tR = 13.4 min, detected at 254 nm; (D) Compound D, tR = 14.7 min, detected at 280 nm; and (E) Compound E, tR = 17.8 min, detected at 254 nm. HPLC analysis was performed using an Agilent 1260 HPLC system equipped with a ZORBAX SB-C18 column (4.6 × 250

mm, 5 μm). Isocratic elution was performed using acetonitrile and 0.1% formic acid in water as the mobile phases. The mobile-phase compositions (acetonitrile/0.1% formic acid in water, v/v) were 33:67 for compounds A and B, 35:65 for compound C, 22:78 for compound D, and 60:40 for compound E. The flow rate was 2.0 mL/min, and the injection volume was 20 μL.
